# Meta-Analysis of Percutaneous Endomyocardial Cell Therapy in Patients with Ischemic Heart Failure by Combination of Individual Patient Data (IPD) of ACCRUE and Publication-Based Aggregate Data

**DOI:** 10.3390/jcm11113205

**Published:** 2022-06-04

**Authors:** Mariann Gyöngyösi, Evgeny Pokushalov, Aleksander Romanov, Emerson Perin, Joshua M. Hare, Jens Kastrup, Francisco Fernández-Avilés, Ricardo Sanz-Ruiz, Anthony Mathur, Wojcieh Wojakowski, Enca Martin-Rendon, Noemi Pavo, Imre J. Pavo, Rayyan Hemetsberger, Denise Traxler, Andreas Spannbauer, Paul M. Haller

**Affiliations:** 1 Division of Cardiology, Department of Internal Medicine II, Medical University of Vienna, 1090 Vienna, Austria; noemi.pavo@meduniwien.ac.at (N.P.); ryan_hemetsberger@hotmail.com (R.H.); denise.traxler-weidenauer@meduniwien.ac.at (D.T.); andreas.spannbauer@meduniwien.ac.at (A.S.); 2 Center of the New and Modern Medical Technologies, 630090 Novosibirsk, Russia; e.pokushalov@gmail.com; 3 E. Meshalkin National Medical Research Center, 630055 Novosibirsk, Russia; abromanov@mail.ru; 4 Stem Cell Center and Adult Cardiology, Texas Heart Institute, Houston, TX 37660, USA; eperin@texasheart.org; 5 Interdisciplinary Stem Cell Institute, Cardiovascular Division, University of Miami Miller School of Medicine, Miami, FL 33136, USA; jhare@med.miami.edu; 6 Cardiology Stem Cell Centre, The Centre for Cardiac, Vascular, Pulmonary and Infectious Diseases, Copenhagen University Hospital Rigshospitalet, 2100 Copenhagen, Denmark; jens.kastrup@rh.regionh.dk; 7 CIBERCV, Instituto de Salud Carlos III, 28029 Madrid, Spain; faviles@secardiologia.es (F.F.-A.); rsanzruiz@hotmail.com (R.S.-R.); 8 Centre for Cardiovascular Medicine and Devices, William Harvey Research Institute, Queen Mary University of London, London EC1M 6BQ, UK; a.mathur@qmul.ac.uk; 9 Department of Cardiology and Structural Heart Diseases, Medical University of Silesia, 40-635 Katowice, Poland; wojtek.wojakowski@gmail.com; 10 R&D Division, National Health Service (NHS)-Blood and Transplant, Oxford Centre, Oxford OX3 9DU, UK; encamartinrendon@gmail.com; 11 Department of Pediatrics, Medical University of Vienna, 1090 Vienna, Austria; imrepavo@gmail.com; 12 Department of Cardiology, University Heart and Vascular Center UKE Hamburg, 20246 Hamburg, Germany; paul.m.haller@gmail.com

**Keywords:** cell-based regeneration therapy, stem cells, ACCRUE, percutaneous transendocardial cell delivery, human clinical trials, meta-analysis

## Abstract

Individual patient data (IPD)-based meta-analysis (ACCRUE, meta-analysis of cell-based cardiac studies, NCT01098591) revealed an insufficient effect of intracoronary cell-based therapy in acute myocardial infarction. Patients with ischemic heart failure (iHF) have been treated with reparative cells using percutaneous endocardial, surgical, transvenous or intracoronary cell delivery methods, with variable effects in small randomized or cohort studies. The objective of this meta-analysis was to investigate the safety and efficacy of percutaneous transendocardial cell therapy in patients with iHF. Two investigators extracted the data. Individual patient data (IPD) (*n* = 8 studies) and publication-based (*n* = 10 studies) aggregate data were combined for the meta-analysis, including patients (*n* = 1715) with chronic iHF. The data are reported in accordance with PRISMA guidelines. The primary safety and efficacy endpoints were all-cause mortality and changes in global ejection fraction. The secondary safety and efficacy endpoints were major adverse events, hospitalization and changes in end-diastolic and end-systolic volumes. Post hoc analyses were performed using the IPD of eight studies to find predictive factors for treatment safety and efficacy. Cell therapy was significantly (*p* < 0.001) in favor of survival, major adverse events and hospitalization during follow-up. A forest plot analysis showed that cell therapy presents a significant benefit of increasing ejection fraction with a mean change of 2.51% (95% CI: 0.48; 4.54) between groups and of significantly decreasing end-systolic volume. The analysis of IPD data showed an improvement in the NYHA and CCS classes. Cell therapy significantly decreased the end-systolic volume in male patients; in patients with diabetes mellitus, hypertension or hyperlipidemia; and in those with previous myocardial infarction and baseline ejection fraction ≤ 45%. The catheter-based transendocardial delivery of regenerative cells proved to be safe and effective for improving mortality and cardiac performance. The greatest benefit was observed in male patients with significant atherosclerotic co-morbidities.

## 1. Introduction

The ACCRUE (meta-analysis of cell-based cardiac studies, NCT01098591) multinational collaborative database was formed to facilitate the investigation of the safety and efficacy of cell-based therapies at an individual patient data (IPD) level, the summarizing harmonized IPDs of cardiac cell therapy studies. The general aim of the ACCRUE IPD-based meta-analysis and the criteria for participating in the database were published in detail previously [1].

According to the equivocal results of cell-based cardiac repair studies and recent meta-analyses [1,2,3], the recent publication on an ineffective intracoronary stem cell trial for patients with acute myocardial infarction (AMI) [4] and the unveiling of research data misuse [5,6,7], the interest of the scientific community in cell-based cardiac regeneration dramatically decreased worldwide with the stop of new intracoronary stem cell trials for patients with AMI.

However, as the number of aging patients with ischemic heart failure (HF) increases worldwide, this population needs an option for new and effective cardiac regenerative therapy on top of the recommended medical treatment. Due to the very limited self-regenerative capacity of adult myocardial cells [8], acute or chronic ischemia is associated with the maladaptive remodeling of cardiac tissue, ultimately leading to HF with reduced ejection fraction (HFrEF). Cell therapy trials of HFrEF patients have aimed to improve cardiac function due to its diverse mechanisms, such as immunomodulation or paracrine or direct effects on the hosting injured myocardial cells. The direct surgical or percutaneous 3D-guided intramyocardial delivery of regenerative medical products have the advantage of the precise application of the substances into the ischemic area, in contrast with the intracoronary catheter-based unselective cell delivery with the partial retention of the cells. However, all intramyocardial cell-therapy trials are relatively small, limiting the statistical power of the outcome analyses. Accordingly, meta-analyses of cardiac regeneration therapies including patients with HFrEF are necessary.

In contrast with trial-based meta-analyses, IPD-based meta-analyses have the ultimate benefit of harmonizing patient baseline and follow-up data, and assessing time-dependent clinical events as well as patient characteristics for predicting clinical and functional outcomes [3]. The first IPD-based meta-analysis summarizing 12 intracoronary stem cell treatment trials including patients with AMI led to the disappointing neutral results of all primary and secondary safety and efficacy endpoints [1].

A major disadvantage of the IPD-based meta-analysis is the dependence on the type and number of recruited studies. Several clinical trials undergo further subanalyses after the publication of the first major results and are, therefore, not ready to provide IPD study data. Recognizing that only 30% of data of percutaneous intramyocardial delivery studies have been included in the ACCRUE (ACCRUE-IPD: MYSTAR, ESCAPE, FOCUS-HF, FOCUS-CCTRN, PRECISE, TAC-HFT, MSC-HF and RENERATE-IHD) [9,10,11,12,13,14,15,16], we combined the IPD-based dataset with the publication-based data (C-Cure, CHART-1, CAUSMIC, MESBLAST-2, SEISMIC, MARVEL, IXMYELOCEL-T, CCTRN-CONCERT-HF Lead-in, CCTRN-CONCERT-HF and DREAM-HF) [17,18,19,20,21,22,23,24,25] and included all percutaneous endomyocardial delivery of cell studies in patients with congestive HF published to date, regardless of cell and delivery catheter types, inclusion criteria, clinical outcomes with diverse definitions of primary and secondary endpoints, and different follow-up times. Hence, the heterogeneity of the data increased but the analysis results became robust, including 18 studies with data of 1715 patients. Interestingly, the primary and secondary outcomes were similar for the “only ACCRUE-IPD” and “combined ACCRUE-IPD and publication-based” meta-analysis results. This finding encouraged us to use the ACCRUE-IPD results to calculate patient characteristics in order to predict the benefits of percutaneous transendocardial cell therapy, the analysis of which requires IPD.

Here, we present the combined IPD and publication-based meta-analysis focused on the percutaneous endomyocardial cell-therapy for patients with chronic HFrEF.

## 2. Methods

### 2.1. Search Strategy

**Figure 1:**
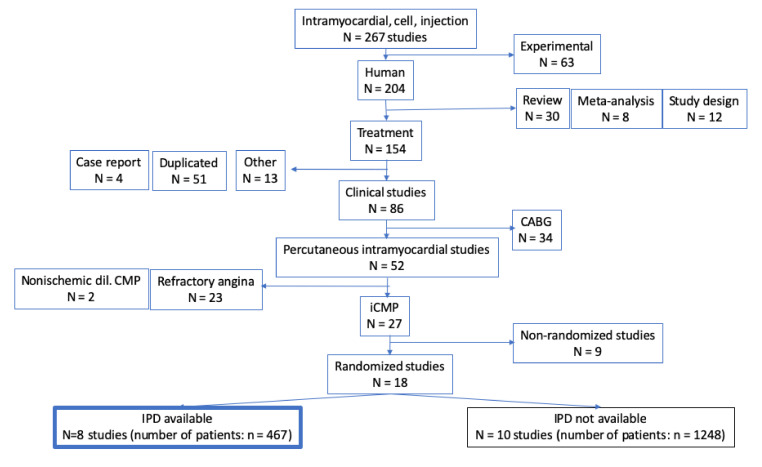
Search strategies: excluded and included studies.

A literature search was repeatedly conducted on the main databases of PubMed, Medline and EMBASE to prospectively include further eligible studies (Figure 1). A further description of the search strategies is included in the Supplementary Methods.

The remaining 18 randomized studies were included. The primary investigators of 8 studies provided full IPDs (MYSTAR, ESCAPE, FOCUS-HF, FOCUS-CCTRN, PRECISE, TAC-HFT, MSC-HF and REGENERATE-IHD) [9,10,11,12,13,14,15,16]. The publication-based aggregate data of the remaining 10 studies (C-CURE, CHART-1, CAUSMIC, MESOBLAST-2, SEISMIC, MARVEL, IXMYELOCEL-T, CCTRN-CONCERT-HF Lead-in, CCTRN-CONCERT-HF and DREAM-HF) were included in the combined meta-analysis to increase the robustness of the general statements (Table 1) [17,18,19,20,21,22,23,24,25]. Supplementary Table S1 provides a quality assessment of the included studies.

### 2.2. Data Collection and IPD Management of the ACCRUE Database

A detailed description of data management in the ACCRUE database was first published 2015 [1]. A brief description is included in the Supplementary Methods.

### 2.3. Inclusion and Exclusion Criteria

We included randomized placebo-controlled cell-therapy studies including patients with HFrEF. Patients were randomized into groups treated either with 3D electromechanical guided (NOGA) or other percutaneous endomyocardial (synonymous intramyocardial or transendocardial) catheter systems (retention-enhanced C-Cathez^TM^; Celyad, Mont-Saint-Guibert, Belgium in CHART-1 and MyoCath^TM^; Bioheart, Inc. of Sunrise, FL, USA in SEISMIC studies) for cell delivery or maximal medical treatment with/without placebo or sham intervention. The exclusion criteria were gene therapy; intracoronary, venous or transvenous sinus coronaries delivery of cells; or surgical direct intramyocardial injections and non-randomized studies.

### 2.4. Primary and Secondary Endpoints Including All Studies

The primary safety endpoint was mortality during follow-up. The primary efficacy endpoint was the changes in left ventricular (LV) EF measured by any imaging modality.

Secondary safety outcomes included major adverse cardiac and cerebrovascular events (MACE), combining mortality, AMI, stroke or any adverse events defined in the studies as a safety endpoint parameter. The secondary efficacy parameter included changes in end-systolic (ESV) and end-diastolic volumes (EDV).

The follow-up of the studies was heterogenous, ranging from 3 to 36 months. The data of different primary efficacy endpoints could not be harmonized to one unique outcome parameter, as described previously [3].

Pooled data of the 3 treatment groups of the dose-escalation MESOBLAST-2 study were recalculated and added to the statistical analysis [20]. Due to the cross-over of patients who were randomized to cell therapy but did not fulfil the inclusion criteria of bone marrow quality in C-CURE and CHART-1 studies, different outcome parameters (e.g., all-cause mortality) were interpreted in intention-to-treat and real treatment groups, resulting in some discrepancies in text and tabulated data of the original publications. As a compromise, the data presented in summary tables of the publications were included in the present analysis [17,18]. The CHART-1 study primary efficacy endpoint was a combined outcome calculated from 6 different parameters [18]. Therefore, the serious adverse events reported by blinded investigators were considered major adverse events (secondary safety endpoint). The CCTRN-CONCERT-HF Lead-in and the following multicenter CCTRN-CONCERT-HF studies were summarized in one paper. To enable us to enter both the primary and secondary endpoints, we separated the 2 studies. The pooled data of the 3 treatment groups of CCTRN-CONCERT-HF were recalculated for one treatment group [24]. The first clinical safety results of the DREAM-HF study were recently reported at the Scientific Session of the American Heart Association 2021 [25].

For primary and secondary efficacy endpoints, the recalculated data of changes in EF (mean ± standard deviation/SD/), EDV and ESV were entered for the C-CURE study. The recalculated SD was entered for the EF changes in SEISMIC study. Due to unpublished left ventricular function data, changes in EF, EDV or ESV from the baseline to follow-up of the CAUSMIC, CHART-1, DREAM-HF, Ixmyelocel-T and MARVEL studies could not be entered into the analysis.

### 2.5. Primary and Secondary Endpoints of ACCRUE IPD Studies

The primary and secondary endpoints of the ACCRUE IPD are included in the Supplementary Methods.

### 2.6. Statistics

#### 2.6.1. Combined Analysis Including Harmonized IPD and Aggregate Data

General meta-analysis rules of the Cochrane Handbook for Systematic Reviews of Intervention (Version 6.2, 2021) were applied (https://training.cochrane.org/handbook/current, accessed on 1 January 2020).

The recalculation of SDs from 95% intervals was performed in accordance with pre-defined formulas (https://training.cochrane.org/handbook/current, accessed on 1 January 2020). Data were pooled and averaged for studies including more than one treatment group [26].

Dichotomous variables were reported as the percentage of the cohort and were compared using a Chi-square test. Continuous parameters are expressed as the mean ± (SD).

Forrest plots of the primary and secondary endpoints were calculated and displayed. Heterogeneity was tested through the application of Cochrane Q statistics and was characterized by I2 values. Sensitivity analysis was used to investigate the influence of separate studies on the outcome.

The IPD of 8 studies allowed for several subgroup analyses, such as Kaplan–Meier survival and event-free survival, Cox regression, to search for predictive factors of the outcome, the correlation between baseline left ventricular functional parameters and their changes, and the effect of the number of cells injected on the outcome.

#### 2.6.2. Analysis of IPD

The statistical methods used for IPD-based meta-analysis were published previously [1]. A brief description is included in the Supplementary Materials.

All statistical computations were performed using Review Manager 5.3 (The Nordic Cochrane Center, Købehvn, Denmark) and Stata/SE, version 12, for Windows (StataCorp, Houston, TX, USA) or R 3.4.2.

## 3. Results

### 3.1. Characteristics of Studies Included in the Analysis

Figure 1 displays the results of the search strategies.

Table 1 summarizes the patient characteristics of all studies.

In total, 1715 patients (with 467 IPD) derived from 18 randomized-controlled trials were included (ACCRUE-IPD: MYSTAR, ESCAPE, FOCUS-HF, FOCUS-CCTRN, PRECISE, TAC-HFT, MSC-HF, and RENERATE-IHD; publication-based: C-Cure, CHART-1, CAUSMIC, MESBLAST-2, SEISMIC, MARVEL, IXMYELOCEL-T, CCTRN-CONCERT-HF Lead-in, CCTRN-CONCERT-HF and DREAM-HF) [9,10,11,12,13,14,15,16,17,18,19,20,21,22,23,24,25]. Supplementary Table S1 displays the quality assessment, and Supplementary Figure S1 exhibits the risk of bias summary of the individual studies included.

Most studies used autologous cells, and two studies (MESOBLAST-2 and DREAM-HF) used allogeneic BM-derived mesenchymal precursor cells, which were delivered intramyocardially. Autologous bone-marrow-derived unselected mononuclear cells (MNCs) were endomyocardially delivered in five studies, mesenchymal stem cells (MSCs) were harvested from the bone marrow (BM) in two studies, adipose-derived regenerative cells were analyzed in one study, either BM-MNCs or BM-MSCs were assessed in one study, BM-derived cardiopoietic cells were studied in two studies, autologous skeletal myoblasts were observed in two studies, and combined BM-origin mesenchymal stromal cells and c-kit positive cells were analyzed in in three studies. Different imaging modalities were used to calculate the LV functional parameter: the early MYSTAR study used single photon emission computed tomography (SPECT), and the majority of later studies evaluated the LV function using echocardiography or magnetic resonance imaging (MRI).

There were short and longer follow-ups in several studies. By harmonizing the IPD of the ACCRUE database to 1-year follow-up, we were able to filter the adverse events during the 12 months and set the maximal follow-up of the IPD meta-analysis at 12 months, which in turn resulted in minor discrepancies in the published data and IPD analysis (e.g., cardiac event after 12 months was not considered in the IPD 1-year follow-up). In contrast, publication-based outcome data of the other 10 studies were reported after predefined follow-up in the range of 3 to 36 months, without the possibility of harmonizing the data to a unique control period.

The majority of the control patients received maximal medical treatment, while the remaining patients received endomyocardial placebo injections.

### 3.2. Patient Characteristics

The baseline patient characteristics were similar in the the cell-therapy and control groups (Table 2), although there were significantly more smokers and a higher incidence of previous percutaneous coronary intervention in the cell-treated group. A significantly higher number of patients carried an automatic implantable cardioverter device (AICD) in the cell therapy group because it was a prerequisite inclusion criterion in several studies, especially in planned autologous skeletal myoblast with known arrhythmogenic potency.

Supplementary Table S2 lists the ACCRUE-IPD baseline data, the results of which are similar to the combined data. Additionally, using the available IPD of some continuous parameters, such as age, the Canadian Cardiovascular Society grading of angina pectoris (CCS), and the New York Heart Association (NYHA) Functional Classification of heart failure, the numbers of delivered cells and the mean or median of these values could be calculated for the randomized patients in the ACCRUE-IPD submeta-analysis. Patients randomized to cell therapy received a median number of cells of 100 × 10^6^ (inter-quartal range (IQR): 42; 100 × 10^6^), administered by a median of 13 (IQR 10;15) injections.

### 3.3. Procedural and In-Hospital Complications

Table 3 provides insights into the procedural, in-hospital and 1-year follow-up events. Most procedural complications were observed in the CHART-1 study using fluoroscopy-guided C-Cath cell delivery. There was a significant benefit to using the 3D NOGA-guided cell delivery compared with the non-NOGA cell delivery systems regarding procedural complications (NOGA: 12/740 patients, 1.6%; vs. other systems: 15/311, 4.8%, *p* = 0.005).

Apart from the notably higher incidence of malignant ventricular arrhythmias in trials using autologous skeletal myoblasts [19,21,22], which also required intensive prophylactical medical treatment, and somewhat more ventricular tachycardias in the cardiopoietic treatment group [18], no short or long-term cell-linked side effects were documented. Significantly higher procedural complications were recorded if the C-Cath or MyoCath transendocardial catheter systems were used, mirroring the definite advantage of NOGA 3D navigation system use. The collection of cells requires invasive procedures, either bone marrow aspiration from the iliac crest, liposuction, muscle biopsy or cardiac biopsy, with the additional risk of local complications. Apart from myocardial perforation in one patient by biopsy, no cell harvesting complications were reported.

### 3.4. Primary and Secondary Clinical Safety Endpoint Analyses

**Figure 2:**
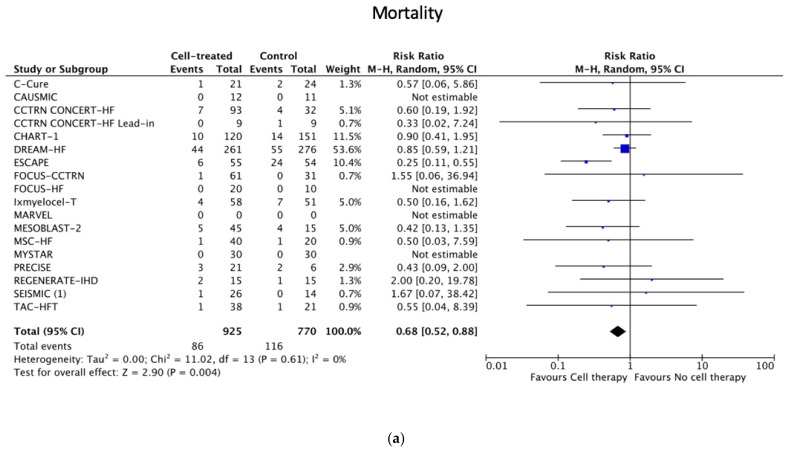

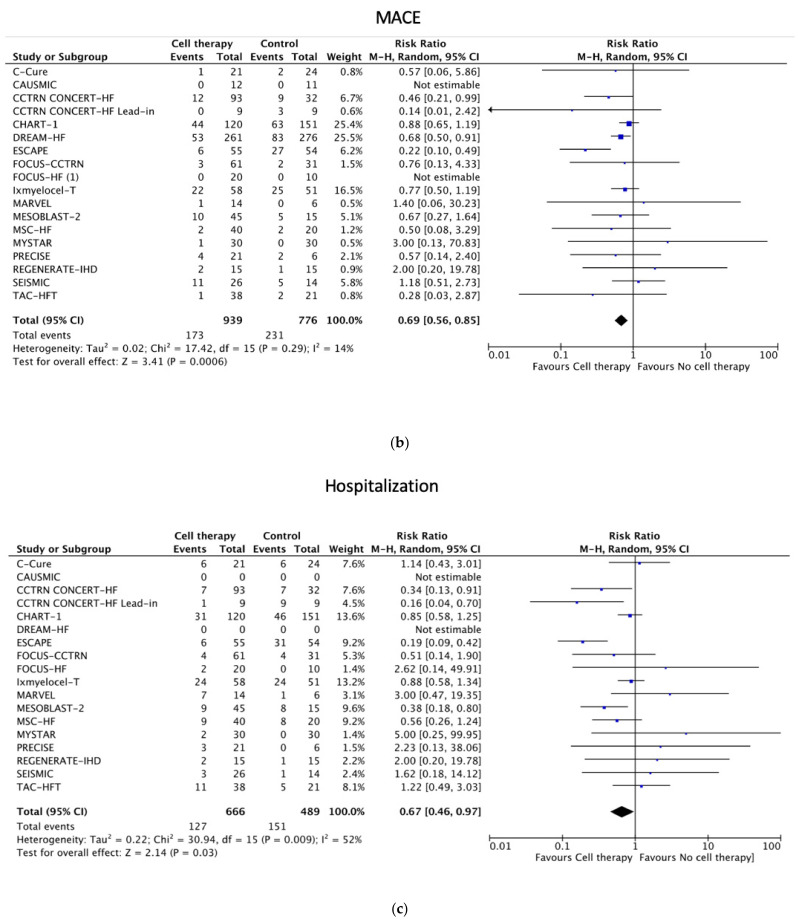
(**a**–**c**). Primary and secondary safety endpoints: clinical outcomes of the studies including patients randomized to receive percutaneous endocardial delivery of regenerative cells or controls. (**a**) Primary safety endpoint: all-cause mortality in patients randomized to receive percutaneous endocardial delivery of regenerative cells or controls. (**b**) Secondary safety endpoint MACEs (major adverse cardiac events) in patients randomized to receive percutaneous endocardial delivery of regenerative cells or controls. (**c**) Secondary safety endpoint: hospitalization in patients randomized to receive percutaneous endocardial delivery of regenerative cells or controls.

Cell therapy showed a significant (*p* < 0.001) reduction in all-cause mortality (primary endpoint) (Figure 2a), MACEs (Figure 2b), hospitalization (Figure 2c) (secondary endpoints) and the incidence of heart transplantation with/without previous implantation of a left ventricular assist device (LVAD), favoring cell therapy during the predefined follow-up in all (*n* = 1715) patients from 18 studies (Table 3). The ESCAPE, CHART-1, DREAM-HF and IXMYELOCEL-T trials presented a relatively high mortality rate, with a consequently high incidence of MACEs and hospitalization. It is noteworthy that the definitions of a serious adverse event were different among the studies. The stepwise exclusion of these studies (sensitivity analysis) did not influence the results.

Owing to the harmonized IPD from 476 patients from eight studies in the ACCRUE-IPD database, we could illustrate cumulative survival, event-free survival curves (Supplementary Figure S2a–c) and Cox regression analyses. The Kaplan–Meier curves confirmed the significant benefit of cell therapy in the ACCRUE-IPD subpopulation, which is in line with the meta-analysis results of the combined data. A Cox regression of the ACCRUE-IPD patients revealed randomization to the control group as the only significant predictor for all primary and secondary safety endpoints.

The incidence of acute myocardial infarction (AMI), stroke, coronary revascularization, and pacemaker (PM) or AICD (with/without cardiac resynchronization therapy/CRT) use was low, and similar results were shown in both groups (Table 3).

The incidence of non-serious adverse events, which did not require additional hospitalization (detection of ventricular thrombus, paroxysmal atrial fibrillation, pneumonia, angina pectoris, etc.) was significantly lower in the cell-therapy group compared with the controls.

Supplementary Table S3 displays similar outcome results to the ACCRUE-IPD subpopulation.

### 3.5. Primary Efficacy Endpoint Outcome

**Figure 3:**
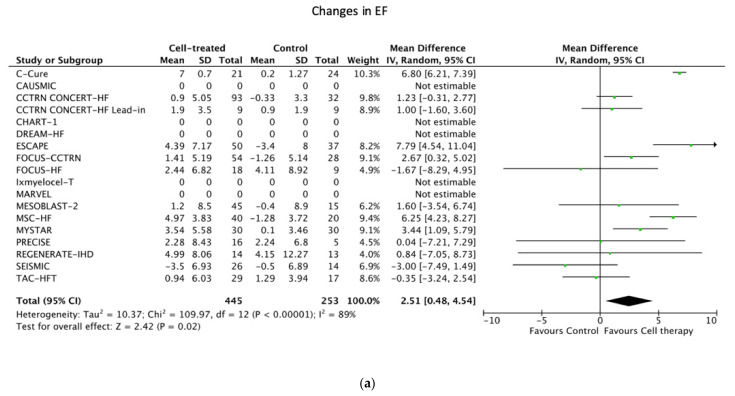

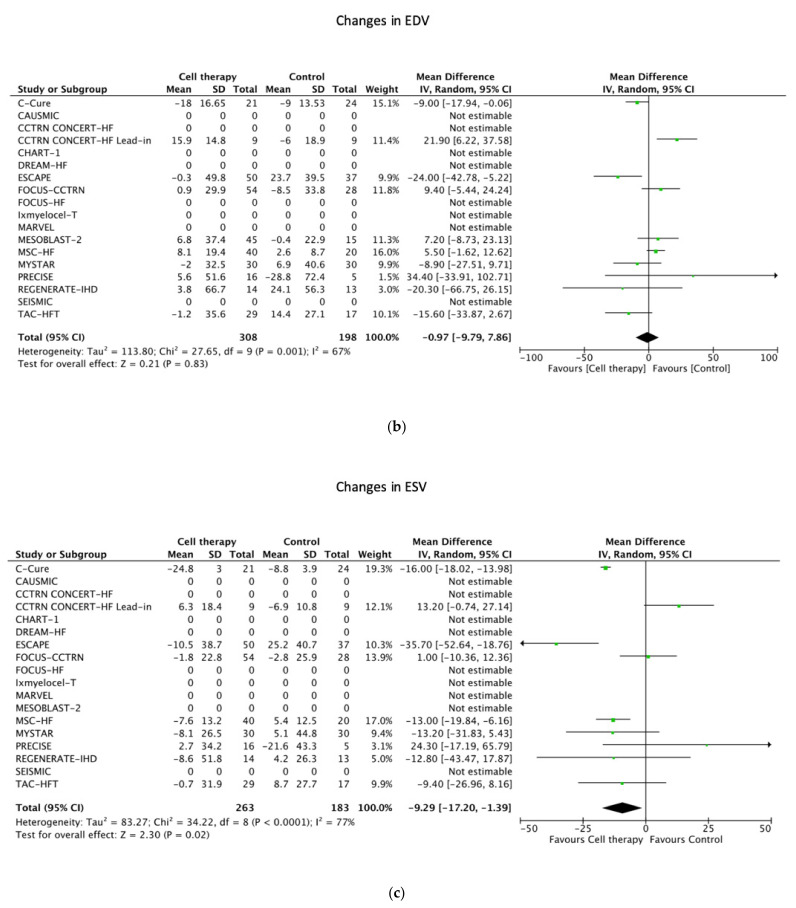
(**a**) Primary efficacy endpoint: changes in EF in patients randomized to receive percutaneous endocardial delivery of regenerative cells or controls. (**b**) Secondary efficacy endpoint: changes in EDV in patients randomized to receive percutaneous endocardial delivery of regenerative cells or controls. (**c**) Secondary efficacy endpoint: changes in ESV in patients randomized to receive percutaneous endocardial delivery of regenerative cells or controls.

Cell therapy led to a significant increase in LVEF, with a mean of 2.51% (95% CI of 0.48; 4.54) both in the combined (*n* = 18 studies) (Figure 3a) and in the ACCRUE-IPD (mean change of 3.1% with a 95% CI of 0.85; 5.34) (*n* = 8 studies) (Supplementary Figure S3a) meta-analysis.

### 3.6. Secondary Efficacy Outcome Results

No meaningful changes in EDV were observed between the groups, neither in the combined nor in the ACCRUE-IPD analyses (Figure 3b and Figure S3b). However, cell therapy led to a significant decrease in ESV in the cell-therapy group (Figure 3b and Supplementary Figure S3b).

### 3.7. Subanalyses of ACCRUE-IPD

The IPD of the ACCRUE-IPD with individual values of the LV functional parameter allowed us to calculate the baseline and follow-up EF, EDV and ESV (Table 4), and the association between the applied cell number; baseline functional data; and changes in EF, EDV and ESV.

The mean LVEF at baseline was somewhat lower in the control patients compared with the cell-treated ones, while there was no difference regarding ESV and EDV (Table 4).

**Figure 4:**
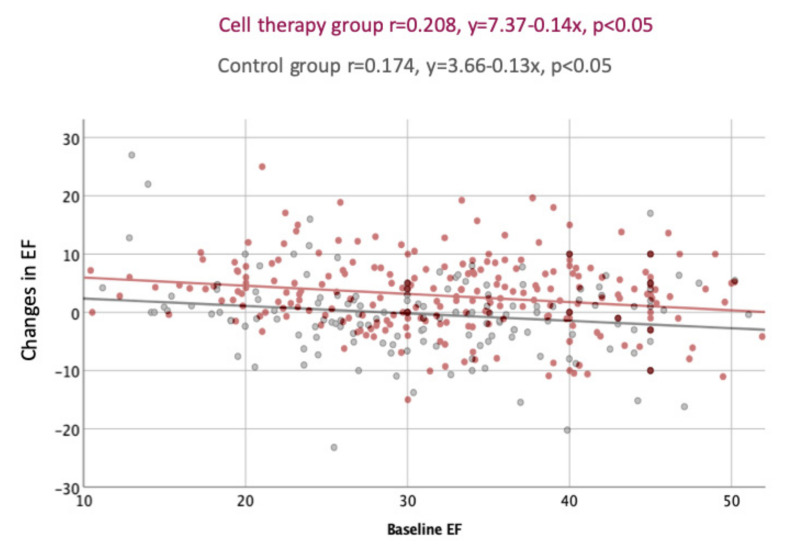
Association between baseline EF and changes in EF including only the IPD of the ACCRUE studies (*n* = 8).

The correlation between baseline EF and changes in LVEF at FUP showed a significant association between lower baseline EF and the improvement of EF at 1-year FUP (delta EF) in both groups, with no difference between the groups (Figure 4).

Dividing the patients according to their baseline EF (Supplementary Table S4), a total of 44.7% of patients had a baseline EF < 30%, indicating a patient collective with severe HFrEF in both groups.

**Figure 5:**
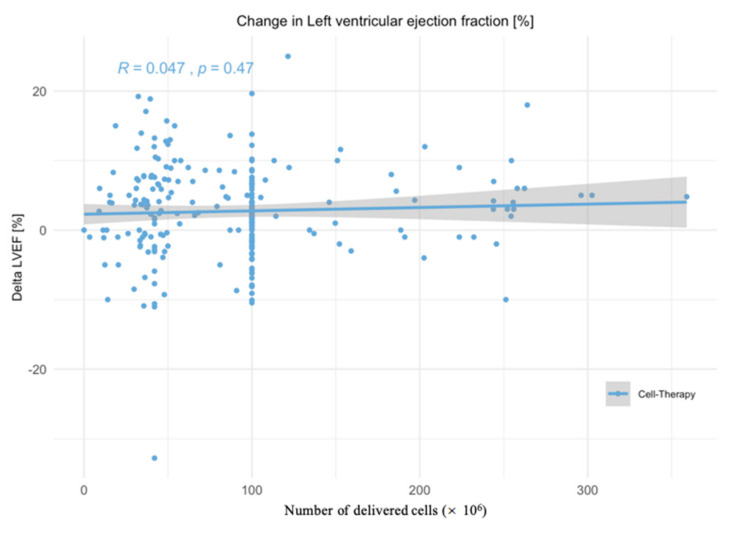
No correlation between number of injected cells and changes in left ventricular ejection fraction (LVEF) at follow-up only IPD of the ACCRUE studies included.

The number of injected cells did not influence the changes in EF (Figure 5).

Plotting the correlation between the baseline EDV and delta-EDV, or ESV and delta-ESV, no significant association could be found (Supplementary Figure S4).

The NYHA classification decreased in both groups but more in the cell therapy group, resulting in a significant difference between the groups (Supplementary Figure S5). The CCS score remained the same in the controls but decreased in the cell-treated patients (Supplementary Figure S5).

Significant weak negative correlation between baseline EF and changes in EF in both groups.

### 3.8. Subanalysis of the Cardiac Function Parameter with Risk Factors Using IPD of ACCRUE Percutaneous Endomyocardial Data

The ANCOVA results are shown in Table 5a (changes in EF), Table 5b (changes in EDV) and Table 5c (changes in ESV). Male patients and those with hyperlipidemia (treated) experienced significant benefits from cell therapy compared with patients with similar characteristics in the control group.

Cell therapy significantly decreased the ESV in male patients; in patients with diabetes mellitus, hypertension or hyperlipidemia; and in those with previous AMI as well as if the baseline EF was ≤45%.

## 4. Discussion

Our meta-analysis on patients with ischemic HFrEF and randomized to receive percutaneous endomyocardial cell therapy showed that percutaneous endomyocardial cell therapy was (1) safe with low procedural complications; (2) associated with a significantly lower rate of mortality, combined major adverse events and less hospitalization; and (3) associated with a significant but small increase in delta EF and a decrease in LV ESV during follow-up. Additionally, an IPD analysis of the ACCRUE subpopulation showed (4) improved NYHA and CCS classification during follow-up and that (5) lower baseline EF was associated with increased improvement in LVEF at follow-up in both the cell-treated and control groups; (6) male patients with high cardiovascular risk profiles (diabetes mellitus, hypertension, hyperlipidemia and previous myocardial infarction) benefitted from cell-based therapies in terms of reduced ESV; (7) the baseline EDV and ESV values did not influence the changes in EDV and ESV during FUP; and (8) the number of cells injected was not correlated with better LVF.

### 4.1. Proposed Mechanisms of the Different Cell Types

Table 6 recapitulates the proposed mechanisms of the applied cell types. All types of cells release paracrine factors, facilitating the immunomodulation of cardiac regeneration on different scales.

### 4.2. Comparison with Published Trial-Based Meta-Analyses including Patients with Severe Heart Failure

Supplementary Table S5 summarizes the results of previous publication-based meta-analyses of ischemic HF trials including cell therapy treatment. Overall, there is a large heterogeneity between the studies, which is mainly due to the inclusion of trials with different cell delivery routes and included patient populations [2,27,28,29,30,31,32,33,34,35,36,37,38,39,40] Direct visual intramyocardial injection during open heart surgery is feasible and offers a relatively simple technology to deliver regenerative substances into the ischemic area, which is not treatable with bypass surgery. However, direct surgical intramyocardial cell delivery studies carry the ethical problems of a proper control group, or the combination of cell delivery and revascularization via aortocoronary bypass operation not enabling the sovereign cell effect. Several meta-analyses on ischemic HF patients also combined studies with the surgical or intracoronary administration of cells, although the intracoronary delivery route is associated with less homing in stem cells [41].

Our combined meta-analysis included patients with severe HFrEF, and we also enrolled patients with non-revascularizable chronic ischemic myocardium. All of the included studies used percutaneous endocardial cell delivery; 16 of the 18 studies used the NOGA 3D electromechanical mapping-guided endocardial injection technique, since this is the only technique that guarantees the on-table visualization of the infarcted area and its border zone. Ultimately, the chronic ischemic myocardium can be delineated with high precision, and the exact positioning of the injection site is feasible, with great accuracy. Additionally, the on-line measurement of the unipolar voltage and local linear shortening values allows for the precise determination of the scar extension and prevents injection into the thinned infarcted wall or injury in the heart conduction system [42].

### 4.3. Analyses of the Primary and Secondary Endpoints

The global assessment of the clinical endpoint events showed a beneficial effect of percutaneous endomyocardial cell therapy on reducing mortality or combined adverse events, or hospitalization.

The ESCAPE, CHART-1, DREAM-HF and IXMYELOCEL-T trials presented relatively high mortality rates, with consequently high incidence of major adverse events and hospitalization, although the definitions of a serious adverse event were different among the studies. The probable reason for the higher adverse event rates of these trials is the more advanced HFrEF, with a high rate of AICD-carrier patients in the trials in DREAM-HF, CHART-1 and IXMYELOCEL-T trials. According to the literature [43,44], the 1-year cardiac mortality of end-stage ischemic HF is up to 15–30%, while the 6 min walking test ≤ 200 m is accompanied by 41% mortality during 40 months of follow-up [45]. In contrast to the patients of the other trials with a low event rate and similarly low EF, the ESCAPE trial enrolled patients between 2005 and 2009, while the majority of the other trials included patients between 2009 and 2019, following new treatment guidelines for HFrEF patients.

The EF improved in the cell-therapy group by a mean of 2.51% compared with the control group (3.1% in ACCRUE-IPD groups), which may not be clinically robust. However, the LV ESV also decreased significantly after cell therapy, considering that the improvement in cardiac function together with the significant decrease in NYHA classification and CCS score is of clinical relevance. It is noteworthy that the mean follow-up EF calculated from the ACCRUE-IPD studies overrode the magic 35% (cut-off of several clinical decisions, such as implantation of a preventive AICD system in ischemic cardiomyopathy) in the cell-therapy group. Control patients had a small but statistically lower EF at baseline. Although a lower EF was significantly associated with an increase in EF at follow-up in both groups, the EF did not change in the ACCRUE-IPD control groups.

Exploiting the advantage of the IPD-based meta-analysis, our subgroup analysis of the comorbidities revealed that male patients and those with high cardiovascular risk profiles benefitted particularly from percutaneous transendocardial cell delivery, which encouraged us to explore the paracrine effect of stem cells in patients with HFrEF. Both the NYHA and CCS classification scores improved significantly in the cell therapy group, indicating clinically important changes in patient well-being.

## 5. Limitations

The study has several limitations, which are included in the Supplementary Discussion section of the Supplementary Materials.

## 6. Conclusions

The percutaneous endomyocardial delivery of different reparative cell types led to a small, clinically non-robust but statistically significant improvement in left ventricular function in patients with HFrEF, with a reduction in mortality, major adverse events and hospitalization, as well as a decrease in NYHA and CCS classification.

## 7. Clinical Implications

While the exact molecular and biological mechanisms of the regenerative cells in the cardiac milieu are still a matter of debate, the angiogenic and myogenic concepts of cardiac regeneration over the last two decades have transferred to paracrine theories, undoubtedly due to the endogen immunomodulatory characteristics of the cells. Although the low engraftment of the externally delivered autologous or allogeneic cells impedes the longevity of the desired effect, regenerative secretory cells can induce epigenetic modulation in “hit-and-run” mode, thereby increasing the paracrine activities in the local molecular environment [46,47]. Our meta-analysis provides insights into clinical trials including HFrEF patients and percutaneous transendocardial regenerative therapies, and patient clinical characteristics to predict outcomes. A broader assessment of the future of clinical cardiac reparative therapy is out of scope of this analysis, and we refer the reader to excellent, recently published overviews on this topic [5,6,34].

## Figures and Tables

**Table 1:** Included studies.

Study Name (Publ. Year)	Study Design	Sample Size (Cell Therapy/Controls)	Inclusion Criteria and Cell Delivery Mode	Cell Type and Amount	Follow-Up Period	Primary Endpoint	Imaging Modality	Intervention Device
**MYSTAR (2009)** [9]	RC, U	30/30	iCMP, HF, pIM	autol. BM-MNCs; 10–13 injection sites; 0.3 mL cells/injection; total volume: 3–3.9 mL; rest injected intracoronary; total cell number: 1.56 ± 0.4 × 10^9^ and 1.55 ± 0.44 × 10^9^	3 m	Changes in infarct size and EF	SPECT	NOGA
**ESCAPE (2010)** [10]	RC, U	55/54	iCMP, HF, pIM	autol. BM-MNCs; 10 injection sites; 0.2 mL/injection; total cell number: 41 ± 16 × 10^6^	12 m	Long-term FUP results	Echo	NOGA
**FOCUS-HF (2011)** [11]	RC, U	20/10	iCMP, HF, pIM	autol. BM-MNCs; 15 injection sites; 0.2 mL cells/injection; total cell number: 100 × 10^6^ cells	6 m	Safety of pIM	Echo	NOGA
**FOCUS-CCTRN (2012)** [12]	RC, MU	61/31	iCMP, HF, pIM	autol. BM-MNCs; 15 injection sites; 0.2 mL/injection; total cell number: 100 × 10^6^ cells	6 m	Change in ESV	Echo	NOGA
**PRECISE (2014)** [13]	RC, MU	21/6	iCMP, HF, pIM	autol. ADRCs; 15 injection sites; total cell number: 42 × 10^6^ cells in 3 mL volume	24 m	Safety and feasibility	MRI	NOGA
**TAC-HFT (2014)** [14]	RC, U	38/21	iCMP, HF, pIM	autol. BM-MSCs or BM-MNCs; 10 injection sites; total cell number: 100 × 10^6^	12 m	30-day SAE	MRI or CT	NOGA
**MSC-HF (2015)** [15]	RC, U	40/20	iCMP, HF, pIM	autol. BM-MSCs; 10–15 injection sites; 0.2 mL/injection; total cell number: 77.5 ± 67.9 × 10^6^	6 m	Change in ESV	MRI or CT	NOGA
**REGENERATE-IHD (2017)** [16]	RC, MU	15/15	iCMP, HF, pIM	autol. BM-MNCs; 10 injection sites; total volume: 2 mL	12 m	Change in EF at 1 y	MRI or CT	NOGA
C-CURE (2013) [17]	RC, MU	21/24	iCMP, HF, pIM	autol. BM-derived cardiopoietic stem cells; mean 18 injection sites; total: 4.5–12.7 mL; mean number of injected cells: 733 × 10^6^	24 m	Feasibility and safety	Echo	NOGA
CHART-1 (2017) [18]	RC, MU	120/151	iCMP, HF, pIM	autol. BM-derived cardiopoietic stem cells; median: 19 injection sites; >24 mio injected cells; median injection volume: 9.6 mL	39 w	Hierarchical composite of 5 safety and efficacy parameters	Echo	C-Cath
CAUSMIC (2009) [19]	RC, MU	12/11	iCMP, HF, pIM	autol. skeletal myoblast; 10 mio cells/injection (0.1 mL in the 30 mio cell dose group, and 0.25 mL in the 25, 100, 300 and 600 mio cell dose group)	12 m	Safety, tolerability and feasibility	Echo	NOGA
MESOBLAST-2 (2015) [20]	RC, MU	45/15	iCMP, noniCMP, HF, pIM	allog. BM-mesenchymal precursor cells; 15–20 injection sites; 0.2 mL/injection (max. 4.0 mL); 25 or 75 or 150 mio MPCs (dose escalating study)	36 m	Safety, tolerability and feasibility	Echo	NOGA
SEISMIC (2011) [21]	RC, MU	26/14	iCMP, HF, pIM	autol. skeletal myoblast; max.: 32 injection sites; 50 mio cells/mL; total: 150–800 mio cells	6 m	Safety: SAE; efficacy: LVEF	MUGA	MyoCath™; Bioheart Inc
MARVEL (2011) [22]	RC, MU	14/6	iCMP, HF, pIM	autol. skeletal myoblast; 16 injection sites; 0.25 mL/injection; total number of cells: 400 × 10^6^ or 800 × 10^6^ cells	6 m	Safety: SAE; efficacy: changes in 6 min WT and MLWHF scores	Echo + MUGA	NOGA
IXMYELOCEL-T (2016) [23]	RC, MU	58/51	iCMP, HF, pIM	autol. BM-origin Ixmyelocel-T; 12–17 injections sites; 0.4 mL/injection; total injection volume: 5.8–8.4 mL	12 m	Composite of 3 safety and combined efficacy parameters	Echo	NOGA
CCTRN-CONCERT-HF Lead-in (2021) [24]	RC, U	9/9	iCMP, HF, pIM	autol. BM-origin MSCs + CPCs; 150 × 10^6^ MSCs and 5 × 10^6^ CPCs	3 m	Safety and feasibility	MRI or CPET	NOGA
CCTRN-CONCERT-HF (2021) [24]	RC, MU	93/32	iCMP, HF, pIM	autol. BM-origin MSCs + CPCs; MSCs: 108 ± 28 × 10^6^ and 4.3 ± CPC: 1.2 × 10^6^;	12 m	Safety, feasibility and efficacy	MRI	NOGA
DREAM-HF (2021) [25]	RC, MU	261/276	iCMP and noniCMP, HF, pIM	allogen. BM-mesenchymal precursor cells	30 m	Recurrent non-fatal decompensated heart failure events per 100 patients	na	NOGA

Bold: IPD available. RC: randomized, controlled; U: unicenter; MU: multicenter; iCMP: ischemic cardiomyopathy; HF: heart failure; BM-MNCs: bone marrow origin mononuclear cells; ADRSs: adipose-derived regenerative cells; BM-MSCs: bone marrow origin mesenchymal stem cells; CPCs: c-kit-positive cells, SPECT: single photon emission tomography; Echo: echocardiography; MRI: magnetic resonance imaging; CT: computed tomography; EF: ejection fraction; ESV: end-systolic volume; FUP: follow-up; SAE: serious adverse event; na: data not available.

**Table 2:** Patient characteristics at baseline.

	Cell Therapy (*n* = 939)	Control (*n* = 776)	*p*-Value
Females	705/930 (12.5%)	113/767 (14.7%)	0.176
Hypertension	705/878 (80.3%)	590/736 (80.2%)	0.950
Hyperlipoproteinemia	413/491 (84.1%)	372/424 (87.7%)	0.129
Diabetes	319/913 (34.9%)	297/762 (27.8%)	0.093
Family history of coronary heart disease	29/108 (26.9%)	20/87 (23.0%)	0.536
Smoking	323/616 (52.4%)	197/460 (42.8%)	0.005
History of myocardial infarction	670/912 (73.5%)	534/761 (70.2%)	0.140
History of coronary artery bypass graft surgery	311/655 (47.5%)	223/485 (57.2%)	0.631
History of percutaneous coronary intervention	476/620 (76.8%)	325/460 (70.7%)	0.025
History of previous AICD	525/853 (61.5%)	348/745 (46.7%)	<0.001
Methods for LV parameter	(*n* = 674)	(*n* = 499)	<0.001
Echocardiography	407 (60.4%)	354 (70.9%)	
MRI	161 (23.9%)	74 (14.8%)	
CT	50 (7.4%)	27 (5.4%)	
SPECT	30 (4.5%)	30 (6.0%)	
MUGA	26 (3.9%)	14 (2.8%)	
Type of cell/placebo			
Autologous BM-MNCs	211 (22.5%)		
Autologous BM-MSCs	59 (6.3%)		
Autologous ADRCs	21 (2.2%)		
Autologous BM-derived cardiopoietic stem cell	141 (15.0%)		
Autologous skeletal myoblast	26 (2.8%)		
Allogeneic BM-mesenchymal precursor cells	261 (27.8%)		
Autologous BM-Ixmyelocel-T	117 (12.5%)		
Autologous BM-MSCs + CPCs	102 (10.9%)		
Placebo		169 (21.8%)	
No injection (max. medical therapy)		607 (78.2%)	

AICD: automatic implantable cardioverter defibrillator; LV: left ventricular; MRI: magnetic resonance imaging; CT: computed tomography; SPECT: single photon emission tomography; BM-MNCs: bone marrow origin mononuclear cells; BM-MSCs: bone marrow origin mesenchymal stem cells; ADRCs: adipose-derived regenerative cells; CPCs: cardiopoietic cells.

**Table 3:** Primary and secondary clinical safety endpoints, complications and adverse events.

All Patients	Cell Therapy (*n* = 939)	Control (*n* = 776)	*p*-Value
Procedural complications	26/611 (4.3%)	1/440 (0.2%)	<0.001
Other in-hospital complications	4/518 (0.8%)	0/408 (0.0%)	0.135
Follow-up events			
Mortality	84/939 (8.9%)	117/776 (15.1%)	<0.001
MACE	173/939 (18.4%)	231/776 (29.8%)	<0.001
AMI	10/570 (1.8%)	3/420 (0.7%)	0.258
Stroke	6/525 (1.1%)	3/405 (0.7%)	0.739
Coronary revascularization	12/450 (2.7%)	5/269 (1.9%)	0.343
Hospitalization	127/666 (19.1%)	151/489 (30.9%)	0.001
HTX or LVAD	8/583 (1.4%)	53/426 (12.4%)	<0.001
PM or AICD Impl.	9/678 (1.3%)	8/500 (1.6%)	0.806
Non-serious AE	94/540 (17.4%)	115/411 (28.0%)	<0.001

MACE, a composite of all-cause death, acute myocardial infarction (AMI), stroke, implantation of left ventricular assist device (LVAD) or heart transplantation (HTX). TVR: target vessel revascularization; PM: pacemaker; AICD: automatic implantable cardioverter defibrillator; AE: adverse event.

**Table 4:** Secondary endpoints: left ventricular baseline (BL) and follow-up (FUP) parameters, based on the ACCRUE-IPD data.

LVF Parameter	Control (*n* = 187)	Cell therapy (*n* = 280)	*p* Value
**EF (%)**			
BL_EF	31.2 ± 9.5	33.2 ± 9.8	0.0341
FUP_EF	31.4 ± 10.5	36.1 ± 10.7	<0.001
Delta EF	−0.4 ± 6.8	2.7 ± 6.7	<0.001
**EDV (mL)**			
BL_EDV	238.6 ± 80.8	235.1 ± 85.1	0.6801
FUP_EDV	247.9 ± 87.7	237.9 ± 90.6	0.3021
Delta EDV	9.2 ± 33.7	2.8 ± 38.4	0.1061
**ESV (mL)**			
BL_ESV	169.3 ± 72.8	162.6 ± 76.2	0.3681
FUP_ESV	176.6 ± 78.3	158.9 ± 80.7	0.0391
Delta ESV	8.3 ± 28.9	−3.8 ± 31.3	< 0.001

BL: baseline; LV: left ventricular; EF: ejection fraction; EDV: end-diastolic volume; ESV: end-systolic volume.

**Table 5:** (**a**) Effect of cell therapy on changes in ejection fraction (EF) in percutaneous cell-therapy and control groups in ACCRUE patients with co-morbidities. (**b**) Effect of cell therapy on changes in end-diastolic volume (EDV) in percutaneous cell-therapy and control groups in ACCRUE patients with co-morbidities. (**c**) Effect of cell therapy on changes in end-systolic volume (ESV) in percutaneous cell-therapy and control groups in ACCRUE patients with co-morbidities.

(a)				
		Changes in EF		
		Mean (SE)	95% CI	*p* value
**Effect of age ≥62**	Cell therapy (*n* = 142)	2.0 (0.7)	+0.6; +3.3	0.195
	Control (*n* = 74)	−0.6 (1.0)	−2.5; +1.2	
**Effect of male gender**	Cell therapy (*n* = 225)	2.1 (0.5)	+1.0; +3.2	<0.001
	Control (*n* = 140)	−1.4 (0.7)	−2.7; −0.1	
**Effect of diabetes mellitus**	Cell therapy (*n* = 65)	1.8 (1.0)	−0.2; +3.8	0.072
	Control (*n* = 48)	−1.7 (1.2)	−4.0; +0.6	
**Effect of hypertension**	Cell therapy (*n* = 206)	1.9 (0.6)	+0.7; +3.0	0.070
	Control (*n* = 128)	−1.4 (0.7)	−2.8; +0.1	
**Effect of hyperlipidemia**	Cell therapy (*n* = 213)	2.4 (0.6)	+1.3; +3.5	0.034
	Control (*n* = 135)	−1.4 (0.7)	−2.8; −0.1	
**Effect of smoking**	Cell therapy (*n* = 145)	1.7 (0.7)	+0.4; +3.0	0.102
	Control (*n* = 79)	−0.1 (0.9)	−1.8; +1.8	
**Effect of baseline EF ≤ 45%**	Cell therapy (*n* = 226)	2.3 (0.5)	+1.2; +3.3	0.151
	Control (*n* = 152)	−0.7 (0.7)	−2.0; +0.6	
(**b**)				
		**Changes in EDV**		
		**Mean (SE)**	**95% CI**	***p*** ** value**
**Effect of age ≥ 62**	Cell therapy (*n* = 129)	3.3 (3.8)	−4.2; +10.9	0.102
	Control (*n* = 69)	9.4 (5.3)	−0.9; +19.8	
**Effect of male gender**	Cell therapy (*n* = 201)	−1.6 (3.1)	−7.7; +4.5	0.732
	Control (*n* = 129)	7.7 (83.9)	−10.5; +31.3	
**Effect of diabetes mellitus**	Cell therapy (*n* = 51)	−7.9 (6.1)	−19.9; +4.1	0.073
	Control (*n* = 40)	0.5 (6.9)	−13.0; +14.1	
**Effect of hypertension**	Cell therapy (*n* = 183)	−0.5 (3.2)	−6.9; +5.8	0.214
	Control (*n* = 116)	9.8 (4.1)	+1.8; +17.8	
**Effect of hyperlipidemia**	Cell therapy (*n* = 191)	−0.56 (3.2)	−6.3; +6.2	0.283
	Control (*n* = 123)	8.1 (4.0)	+0.3; +15.9	
**Effect of smoking**	Cell therapy (*n* = 127)	−4.6 (3.9)	−12.2; +3.1	0.108
	Control (*n* = 72)	3.2 (5.2)	−6.9; +13.3	
**Effect of baseline EF ≤ 45%**	Cell therapy (*n* = 201)	−1.2 (3.1)	−7.3; +5.0	0.152
	Control (*n* = 137)	9.4 (3.8)	+1.0; +16.8	
(**c**)				
		**Changes in ESV**		
		**Mean (SE)**	**95% CI**	***p*** ** value**
**Effect of age ≥62**	Cell therapy (*n* = 129)	−3.3 (3.0)	−9.2; +2.7	0.282
	Control (*n* = 69)	8.0 (4.1)	+0.1; +16.1	
**Effect of male gender**	Cell therapy (*n* = 201)	−7.1 (2.4)	−11.9; −2.3	0.004
	Control (*n* = 129)	8.0 (3.0)	+2.0 +13.9	
**Effect of diabetes mellitus**	Cell therapy (*n* = 51)	−11.2 (4.8)	−20.7; −1.8	0.020
	Control (*n* = 40)	5.7 (5.4)	−5.0; +16.3	
**Effect of hypertension**	Cell therapy (*n* = 183)	−6.1 (2.5)	−11.1; −1.1	0.020
	Control (*n* = 116)	9.6 (3.2)	+3.3; +15.9	
**Effect of hyperlipidemia**	Cell therapy (*n* = 191)	−6.4 (2.5)	−11.3; −1.5	0.011
	Control (*n* = 123)	8.6 (3.1)	+2.5; +14.7	
**Effect of smoking**	Cell therapy (*n* = 127)	−8.7 (3.0)	−14.7; −2.8	0.634
	Control (*n* = 72)	1.2 (4.0)	−6.7; +9.1	
**Effect of baseline EF ≤ 45%**	Cell therapy (*n* = 201)	−7.3 (2.4)	−12.2; −2.5	0.003
	Control (*n* = 137)	8.5 (3.0)	+2.7; +14.3	

**Table 6:** Proposed mechanisms of the different cell types.

Type of Cell	Studies	Proposed Mechanisms
Autologous BM-MNC [9,10,11,12,14,16]	MYSTAR, ESCAPE, FOCUS-HF, FOCUS-CCTRN, REGENERATE-HD, TAC-HFT	Secretion of angiogenic chemokines and cytokines, and ability to recruit cells and promote cell survival; upregulation of endogenous cytokine expression
Autologous BM-MSCs [15]	MSC-HF	Multipotent stem cells, paracrine stimulation of resident cardiac stem cells
Autologous ADRCs [13]	PRECISE	Mixed, multipotent population of cells, differentiating into multiple cell lineages, such as cardiomyocytes, endothelial and smooth muscle cells; secretion of growth factors and cytokines
Autologous BM-derived cardiopoietic stem cell [17,18]	C-CURE, CHART-1	Nuclear translocation of cardiac transcription factors; increase in Nkx2.5, Flk-1, Gata-6, and Fog-1
Autologous skeletal myoblast [19,21,22]	CAUSMIC, SEISMIC, MARVEL	Myogenic phenotype; increase in graft survival; intrinsic resistance to hypoxia; up-regulation of pro-angiogenic, anti-apoptotic, heart development and extracellular matrix remodeling genes; and secretion of growth factors
Allogeneic BM-mesenchymal precursor cells [20,25]	MESOBLAST-2, DREAM-HF	Multipotent nonhematopoietic stem cells, enrichment of the Stro-1/Stro-3+ population of mesenchymal lineage precursors, extensive proliferation, differentiation in vitro into different cell types, secretion of multiple paracrine factors and a decrease in apoptosis
Autologous BM-Ixmyelocel-T [23]	Ixmyelocel-T	Has the regenerative properties of MSCs, but a 200 times higher number of M2 macrophages and 50 times higher number of CD90+ MSCs
Autologous BM-MSCs + CPCs [24]	CCTRN-CONCERT-HF Lead-in, CCTRN-CONCERT-HF	CPCs differentiate into endothelial cells, and release paracrine signals, combining the 2 different cell types results into a complementary impact on secretome production

BM-MNCs: bone marrow mononuclear cells; MSCs: mesenchymal stem cells; ADRCs: adipose-derived regenerative cells; HF: heart failure; CPCs: c-kit positive cardiac cells.

## Data Availability

The data are stored by the study primary investigator (MG) and are available upon reasonable request.

## Supplementary Materials

The following supporting information can be downloaded at https://www.mdpi.com/article/10.3390/jcm11113205/s1, Figure S1. Risk of bias summary. Figure S2a. Clinical primary safety endpoint analyses of the ACCRUE IPD patients: all-cause mortality (*n* = 8 studies). Figure S2b. Clinical safety secondary endpoint analyses of the ACCRUE patients: MACCE (*n* = 8 studies). Figure S2c. Clinical safety secondary endpoint analyses of the ACCRUE patients: hospitalization (*n* = 8 studies). Figure S3a. Left ventricular functional primary efficacy analysis of the ACCRUE patients (*n*=8 studies). Figure S3b. Secondary efficacy endpoint: changes in end-diastolic volume (EDV) of the ACCRUE patients (*n* = 8 studies). Figure S3c. Secondary efficacy endpoint: changes in end-systolic volume (ESV) of the ACCRUE patients (*n* = 8 studies). Figure S4. Association between baseline end-diastolic (EDV) and end-systolic volumes (ESV) and changes in EDV and changes in ESV of the ACCRUE patients. Figure S5. Significant improvement in NYHA and CCS classes during the follow-up of the ACCRUE patients. Table S1. Quality assessment. Table S2. Patient characteristics of ACCRUE-IPD at baseline. Table S3. Primary and secondary clinical safety endpoints, complications and adverse events in the ACCRUE-IPD patients. Table S4. Distribution of baseline ejection fraction (EF), based on ACCRUE IPD data (*n* = 8 ACCRUE studies). Table S5. Summary of heart failure (HF) cell therapy meta-analyses.
