# Supplementary material for: Meta-Analysis of Percutaneous Endomyocardial Cell Therapy in Patients with Ischemic Heart Failure by Combination of Individual Patient Data (IPD) of ACCRUE and Publication-Based Aggregate Data
Source: J Clin Med. 2022 Jun 4;11(11):3205. doi: 10.3390/jcm11113205 (PMC9181462; doi:10.3390/jcm11113205)
Supplement: Supplementary file 1 [file jcm-11-03205-s001.zip › jcm-1733080-supplementary.pdf]

Meta-analysis of percutaneous endomyocardial cell-therapy in patients with ischemic heart failure by combination of Individual Patient Data (IPD) of ACCRUE and publication-based aggregate data.

Supplementary Material

SUPPLEMENTARY METHODS

Search strategies

After exclusion of abstracts and conference papers, 267 studies have been identified, 63 of them were experimental and 204 human clinical papers Reviews, meta-analyses and study design reports were further omitted. Case reports presenting single patients, duplicated publications with different follow-ups, sub-analysis or subgroups of the same original studies were excluded. Other trials reporting pediatric patient treatment or transvenous cell applications, or intramyocardial laser therapies were also excluded. Direct surgical intramyocardial cell applications were reported in 34 trials, and percutaneous catheter-based cell delivery in 52 studies. The indication for percutaneous transendocardial cell therapy was non-ischemic dilated cardiomyopathy, refractory angina aiming at the revascularization of the ischemic myocardium without heart failure symptoms, or ischemic cardiomyopathy resulting in HF, with the objective to improve myocardial function. Among the latter, 9 studies were non-randomized, or open-labeled or dose-finding studies without placebo/medical therapy control group.

Data collection and IPD management of the ACCRUE Database

In brief, primary investigators and corresponding authors of eligible studies were contacted multiple times, via E-Mail or in person, and asked to contribute IPD to the ACCRUE database. For data transfer and data harmonization, a case-report form with predefined terms, conditions and definition, which were agreed on at the first ACCRUE investigator meeting, was used for the deposition of IPDs. Solely depersonalized data were collected, and data transfer was in accordance with institutional regulations. The total number of included patients with available IPDs were 467 (280 and 187 patients randomized to cell therapy vs placebo treatment) (Suppl. Table S2).

Endpoints of the ACCRUE-IPD

The primary safety endpoint of the ACCRUE-IPD was the all-cause mortality in the IPD studies. The primary efficacy endpoint was the changes in LVEF measured by any imaging modality during follow-up of the initial study.

Secondary safety outcomes included combined MACE (a composite of all-cause death, AMI, stroke, implantation of LVAD or heart transplantation) and hospitalization. The secondary efficacy parameter included the ESV and EDV, respectively, the change in cardiac function parameters (delta-ESV, delta-EDV and delta-LVEF), the association between baseline functional parameter and changes during the 1-year follow-up, the association between injected cell number and changes in LVEF and the follow-up NYHA class.

Subgroup analysis comprised the confounding factors predicting the success of the cell-therapy, measured by analysis of covariance (ANCOVA).

The REGENERATE-IHD studies included several groups, therefore we have extracted the data of the patients randomized to percutaneous intramyocardial delivery and the respective control group. In the unique MYSTAR study, there was a cross-randomization of the patients in the "late group" after 3 months, therefore, the data of the first 3 months after randomization were considered only, when the patients in the "late group" still received maximal medical treatment. The MSC and BMC groups were pooled in the TAC-HFT study.

Supplementary statistics

Assessment of risk of bias

Two authors (MG and PH) assessed the risk of bias independently for each study in accordance with the criteria published in the Cochrane Handbook for Systematic Reviews of Intervention. The criteria were grouped to patient selection bias (adequate allocation, method of randomization, and the baseline characteristics of the patients of the separate groups), study performance bias (patient, staff blinded to the study), evaluation bias (blinded analysis) and attrition bias (loss of patients during the follow-up).

Analysis of IPD

Briefly, all IPD analyses were performed based on intention-to-treat.

Continuous parameters were expressed as mean ± (SD) or median with 25%-75% interquartile ranges (IQR) if they were tested for normal or non-normal distribution, respectively. Groups were compared with either unpaired two-sided Student t-test or nonparametric test Mann-Whitney U-test as appropriate. Association between variables was assessed by using linear regression analysis.

IPD Meta-analysis

For the primary endpoint assessment, the time-dependent event rate was calculated and compared between groups by log-rank test, and plotted using Kaplan-Meier curves. Adjustments for patient characteristics were analyzed using Cox regression models providing hazard ratios (HR) with 95% confidence intervals (CI). Standardized mean differences and corresponding 95% CI for scale variables were calculated using random-effects model (if heterogeneity test >50%) and presented with a forest plot.

Subgroup analyses

The treatment effect of the individual patients with pre-defined comorbidities were tested by means of analysis of covariance (ANCOVA). The changes in EF, EDV and ESV were related to male gender, age >62y (median of all patients), presence of diabetes mellitus, hypertension, hyperlipidemia, smoking, positive family history of coronary artery disease, previous myocardial infarction, coronary artery bypass grafting (CABG), or percutaneous coronary intervention (PCI), the imaging modality of cardiac MRI, cell-type or baseline EF≤45%. The mean changes with 95% CIs were reported. All *P* values were based on two-sided tests.

SUPPLEMENTARY DISCUSSION

Limitations

We have combined the IPD and aggregate data to increase the robustness of the analysis, even if the heterogeneity of the analysis increased, especially regarding follow-up time, used cell types and definition of clinical outcomes. Several studies with aggregate data did not report left ventricular functional data. The selection of patients by diverse inclusion (eg. baseline EF, previously implanted AICD) and exclusion criteria led to diversified population in the cell-based therapy trials. In addition, the character of the chosen reparative cells influences the outcomes, even if the main mechanism is attributed to the paracrine machinery of each cell type. This mirrors the continuous attempt to improve the efficiency of the cardiac regenerative therapies for patients with ischemic HF. In spite of intensive research on clinical cardiac regeneration in the last 2 decades, the question on patient characteristics and cell types remains still open. Narrowing the patient`s availability by stronger inclusion criteria led to difficult and slow recruitment rate in several trials, such as the MARVEL with suspension of enrollment.

All studies strived for application the maximal number of cells. However, there is no real consensus how many cells should be delivered. The NOGA injection catheter delivery system allows max. 0.4 mL (but rather 0.2-0.3 mL) injected substances. The used cells are relatively large, and care should be taken, that the cell substance does not plug the catheter internal lumen. Since at least 1 cm between the injection sites is recommended, and 12-20 injections are applied per patient, the number of the injections with the maximal number of cells is limited.

Finally, meta-analyses do not replace multicenter randomized controlled studies, and the large randomized NOGA-guided cell-therapy study, including over 100 patients (SCIENCE) is currently on-going, for which first results are expected in 2022 [S1].

To note, JNJ's Cordis division has withdrawn the NOGA endocardial injection catheter technology, mainly due to business reason. There are several reasons for the withdrawal of the percutaneous intramyocardial delivery systems, one of them is certainly the failure of the expected robust clinical effect, but other, mainly financial causes were considered, such as lack of FDA approval of the system, which is necessary for commercial disposal the system to the US and other non-US study sites, as well as low number of the includable patients with slow recruiting rate, and high costs of the catheter production and also the costs of the

expensive human procedures. Currently, a consortium has been built to try to find a solution also for the on-going-NOGA-guided EU-sponsored trials, and to save the technology offering the only 3D-guided on-table viability measurement and precise catheter-based intramyocardial delivery tool.

Abbreviations

ACCRUE	meta-Analysis of Cell-based CaRdiac stUdiEs
ADRC	Adipose-derived regenerative cell
AE	Adverse event
AICD	Automatic implantable defibrillator
AMI	Acute myocardial infarction
ANCOVA	Analysis of Covariance
BL	Baseline
BM	Bone marrow
CCS	Canadian Cardiovascular Society Angina Grade
CI	Confidence Interval
CPC	Cardiac progenitor cell
CRT	Cardiac resynchronization therapy
CT	Computed tomography
EDV	End-diastolic volume
ESV	End-systolic volume
FUP	Follow-up
HF	Heart failure
HFrEF	Heart failure with reduce ejection fraction
HTX	Heart transplantation
iCMP	Ischemic Cardiomyopathy
iHF	Ischemic Heart Failure
IPD	Individual Patient Data
IQR	Interquartile range
LV	Left ventricular
LVAD	Left ventricular assist device
LVF	Left ventricular function
MACE	Major adverse cariac events
MC	Multicenter
MNC	Mononuclear Cell
MRI	Magentic resonance Imaging
MSC	Mesenchymal Stem Cell
MUGA	Multigated acquisition scan
na	not available
NYHA	New York Heart Failure Association Functional Classification
pIM	Percutaneous intramyocardial delivery

PM	Pacemaker
RC	Randomized controlled
SAE	Serious adverse event
SD	Standard deviation
SPECT	Single Photon Emmission Computed Tomography
U	Unicenter

Supplementary Table S1. Quality assessment

Bias	Selection			Study performance	Evaluation	Attrition
	Adequate allocation	Method adequate for randomization	Groups are similar at the start of the study	Study patients/staffs blinded to study	Study analysis blinded to randomization	Loss to follow-up
**MYSTAR (2009)** [Su, #540]	Yes	Yes	Yes	No	Yes	0%
**ESCAPE (2010)** [10]	Yes	Yes	Yes	Yes	Yes	0%
**FOCUS-HF (2011)** [11]	Yes	Yes	Yes	No	Yes	0%
**FOCUS-CCTRN (2012)** [12]	Yes	Yes	Yes	Yes	Yes	0%
**PRECISE (2014)** [13]	Yes	Yes	Yes	Yes	Yes	0%
**TAC-HFT (2014)** [14]	Yes	Yes	Yes	Yes	Yes	0%
**MSC-HF (2015)** [15]	Yes	Yes	Yes	Yes	Yes	0%
**REGENERATE-IHD (2017)** [16]	Yes	Yes	Yes	Yes	Yes	6.7%
C-CURE (2013) [17]	Yes	Yes	Yes	No	Yes	0%
CHART-1 (2017) [18]	Yes	Yes	Yes	No	Yes	0%
CAUSMIC (2009) [Su, #540]	Yes	Yes	Yes	No	Yes	0%
MESOBLAST-2 (2015) [20]	Yes	Yes	Yes	Yes	Yes	0%
SEISMIC (2011) [21]	Yes	Yes	Yes	No	Yes	0%
MARVEL (2011) [22]	Yes	Yes	Yes	Yes	Yes	0%

IXMYELOCEL-T (2016) [23]	Yes	Yes	Yes	Yes	Yes	0%
CCTRN-CONCERT-HF Lead-in (2021) [24]	Yes	Yes	Yes	No	Yes	0%
CCTRN-CONCERT-HF (2021) [24]	Yes	Yes	Yes	Yes	Yes	0%
DREAM-HF (2021) [25]	Yes	Yes	Yes	Yes	Yes	0%

Bold: IPD available. The references in this table refer to the bibliography in the main text.

Supplementary Table S2. Patient characteristics of ACCRUE-IPD at baseline

	Control (N=187)	Cell-Therapy (N=280)	Total (N=467)	p-value
Age	61.0 (54.5. 67.6)	62.0(54.5. 68.6)	62.0 (54.5. 68.1)	0.265
Females	24/187 (12.8%)	32/280 (11.4%)	56 (12.0%)	0.647
CCS at baseline	3.0 (2.0. 3.0)	2.0 (1.8. 3.0)	3.0 (2.0. 3.0)	0.423
NYHA class	3.0 (2.0. 4.0)	3.0 (2.0. 3.0)	3.0 (2.0. 4.0)	0.020
Hypertension	152/187 (81.3%)	228/280 (81.4%)	380 (81.4%)	0.969
Hyperlipidemia	160/187 (85.6%)	231/280 (82.5%)	391 (83.7%)	0.380
Diabetes	52/187 (27.8%)	72/280 (25.7%)	124 (26.6%)	0.616
Family history of CAD	20/87 (23.0%)	29/108 (26.9%)	49 (25.1%)	0.536
Smoking	88/187 (47.1%)	161/287 (57.9%)	249 (53.5%)	0.021
History of MI	129/167 (77.2%)	196/236 (83.1%)	325 (80.6%)	0.146
History of CABG	107/187 (57.2%)	160/280 (57.1%)	267 (57.2%)	0.987
History of PCI	66/113 (58.4%)	126/175 (72.0%)	192 (66.7%)	0.017
Number of dis-eased coronary vessels	3.0 (1.0. 3.0)	3.0 (1.0. 3.0)	3.0 (1.0. 3.0)	0.907
**Methods for LV parameter**				0.256
Echocardiography	96 (51.6%)	137 (49.6%)	233 (50.4%)	
MRI	33 (17.7%)	59 (21.4%)	92 (19.9%)	
CT	27 (14.5%)	50 (18.1%)	77 (16.7%)	

SPECT	30 (16.1%)	30 (10.9%)	60 (13.0%)	
**Type of cells/placebo**				
BM-MNC		199 (71.1%)		
BM-MSC		59 (21.1%)		
ADRC		21 (7.5%)		
Placebo	103 (55.1%)			
No injection	84 (44.9%)			

One patient randomized to cell therapy received no injection. Values are given as median with interquartile values

CCS: Canadian Cardiovascular Society grading of angina pectoris, NYHA: New York Heart Association (NYHA) Functional Classification of heart failure, CAD: Coronary Artery Disease; MI: Myocardial Infarction, CABG: Coronary Artery Bypass Graft Surgery, PCI: Percutaneous Coronary Intervention, LV: left ventricular, MRI: magnetic resonance imaging, CT: computed tomography, SPECT: single photon emission tomography, BM-MNC: bone marrow origin mononuclear cells, BM-MSC: bone marrow origin mesenchymal stem cells, ADRC: adipose-derived regenerative cells.

Supplementary Table S3. Primary and secondary clinical safety endpoints, complications and adverse events in the ACCRUE-IPD patients

ACCRUE-pIM	Control (N=187)	Cell-Therapy (N=280)	Total (N=467)	p-value
**Procedural complications**	0	5 (1.8%)	5 (1.1%)	0.163
Inhospital other complications	0	1 (0.4%)	1 (0.2%)	1.0
**1-year Follow-up**				
MACCE	34 (18.2%)	19 (6.8%)	53 (11.3%)	<0.001
Mortality	27 (14.4%)	14 (5.0%)	41 (8.8%)	<0.001
AMI	2 (1.1%)	3 (1.1%)	5 (1.1%)	1.0
Stroke	2 (1.1%)	4 (1.4%)	6 (1.3%)	1.0
TVR	1 (0.5%)	1 (0.4%)	2 (0.4%)	1.0
Hospitalization	49 (26.2%)	39 (13.9%)	88 (18.8%)	0.001
HTX or LVAD	3 (1.6%)	2 (0.7%)	5 (1.1%)	1.0
PM or ICD Impl.	8 (4.3%)	5 (1.8%)	13 (2.8%)	0.147
Non-serious AE	20 (10.7%)	21 (7.5%)	41 (8.8%)	0.246

MACCE, a composite of all-cause death, acute myocardial infarction (AMI), stroke, implantation of left ventricular assist device (LVAD) or heart transplantation (HTX), TVR: Target Vessel Revascularization, PM: Pacemaker, ICD: Implantable Cardioverter Defibrillator, AE: adverse event

Supplementary Table S4. Distribution of baseline ejection fraction (EF), based on ACCRUE IPD data (n=8 ACCRUE studies)

Category of baseline EF	Randomized to Control	Randomized to cell therapy	Total number of patients
Baseline EF >50%	3 (1.6%)	10 (3.7%)	13 (2.9%)
Baseline EF >45-≤50%	6 (3.3%)	15 (5.6%)	21 (4.7%)
Baseline EF >40-≤45%	27 (14.8%)	38 (14.2%)	65 (14.4%)
Baseline EF >35-≤40%	25 (13.7%)	46 (17.2%)	71 (15.8%)
Baseline EF >30-≤35%	32 (17.6%)	47 (17.5%	79 (17.6%)
Baseline EF <30%	89 (48.9%)	112 (41.8%)	201 (44.7%)

Baseline EF was not defined in 17 cases. Percentage of the respective group (Control n=182. Cell therapy n=268). No difference between the groups.

Supplementary Table S5. Summary of heart failure (HF) cell therapy meta-analyses.

Studies were excluded if studies included patients with recent acute myocardial infarction.

Publications	Inclusion criteria	No. of studies	Cell-treated / control patients	Delivery route	Mortal-ity	Combined adverse cardiac events	LV EF
Jayaraj et al 2019 [S2]	HF trials be-tween 2017-2019	6	569*	im (percut or surg.). ic. iv	no differ-ence	n.r.	EF im-proved 4.58% (172 vs 195 pts)
Fan et al 2019 [S3]	HF, ICMP, NICMP, MSC	9	320 / 292**	im (percut or surg.). ic. iv	no differ-ence	Hospit. Reduced	EF im-proved 5.25%
Fu et al 2018 [S4]	HF, MSC	6	271 / 254	im (percut or surg.). ic. iv	no differ-ence	no differ-ence	EF im-proved 9.64% (172 vs 195 pts)
Fisher et al 2016 [S5]	HF	38	1114 / 793	im (percut or surg.). ic. iv	reduced	AMI re-duced	no differ-ence
Fisher et al 2015 [S6]	HF, iCM and NICM	31	626 / 895	im (percut or surg.)	reduced	Hospit. Reduced	EF im-proved 4.66%
Tian et al. 2014 [S7]	CIHD	11	272 / 220	im (percut or surg.)	reduced	n.s.	improved 4.91%
Xu et al. 2014 [S8]	CIHD	19	440 / 309	im (percut or surg.)	reduced	n.s.	improved 3.54%

Xiao et al 2014 [S9]	CIHD	19	453 / 322	im (percut or surg.). ic.	n.s.	n.s.	improved 3.35%
Fisher et al. 2014 [S10]	CIHD, HF	23	659 / 478***	im (percut or surg.). ic	reduced	Hospit. Reduced	improved 2.62%
Cheng et al. 2013 [S11]	ICMP	5	135 / 75	im (percut or surg.)	n.s.	NR	no differ-ence
Kandala et al. 2013 [S12]	ICMP	10	283 / 236	im (surg.). ic	n.s.	NR	improved 4.48%
Wen et al. 2012 [S13]	IHD, HF	13	378 / 280	im (percut or surg.). ic	reduced	NR	improved 5.67%
Zhao et al. 2011 [S14]	IHD	10	250 / 207	im (surg.). ic	NR	NR	improved 4.02%
Donndorf et al. 2011 [S15]	IHD	6	94 / 85	im. (surg)	n.s.	n.s.	improved 5.04%

*Different number of studies included in safety and efficacy analyses,

** Non-randomized studies also included

*** EF calculated in 184 vs 126 pts

AMI: acute myocardial infarction; AP: angina pectoris, CABG: coronary artery bypass graft, CIHD: chronic ischemic heart disease, HF: heart failure, ICMP: ischemic cardiomyopathy, NICMP: non-ischemic cardiomy-opathy, MSC: mesenchymal stem cell, i.c.: intracoronary application, i.m.: intramyocardial, n/r.: not reported, n.s.. not significant; NYHA. New York Heart Association; perc. percutaneous; *statistical significant between groups in subgroup analyses.

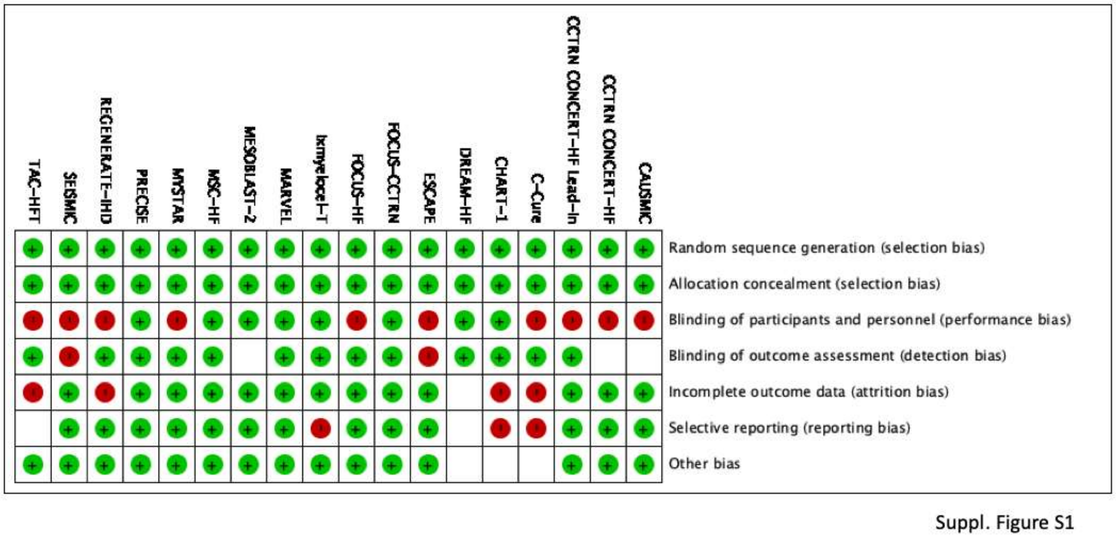

Supplementary Figure S1. Risk of bias summary

A

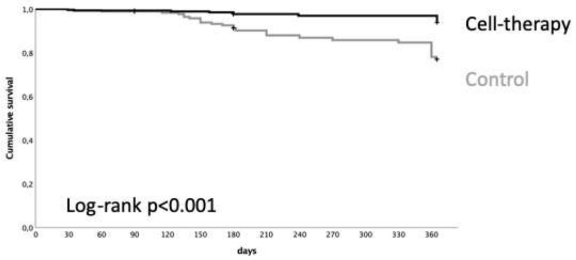

B

All-cause mortality	Control (N=187)	Cell-Therapy (N=280)	Total (N=467)
ESCAPE	24 (44.4%)	6 (10.9%)	30 (27.5%)
FOCUS-CCTRN	0 (0.0%)	1 (1.6%)	1 (1.1%)
FOCUS-HF	0 (0.0%)	0 (0.0%)	0 (0.0%)
MSC-HF	1 (5.0%)	1 (2.5%)	2 (3.3%)
MYSTAR	0 (0.0%)	0 (0.0%)	0 (0.0%)
PRECISE	0 (0.0%)	3 (11.1%)	3 (11.1%)
Regenerate-IHD	1 (6.7%)	2 (13.3%)	3 (10.0%)
TAC-HF	1 (4.8%)	1 (2.6%)	2 (3.4%)

C

	Hazard ratio	95% CI	p value
Age>62y	0.522	0.257; 10.6	0.072
Male gender	1.484	0.452; 4.866	0.515
DM	0.464	0.139; 1.546	0.211
Hypertension	8.151	1.065; 16.24	0.043
Hyperlipidemia	0.971	0.218; 4.333	0.97
Previous MI	0.001	0.512; 2.537	0.957
Baseline EDV>238 mL	1.223	0.616; 2.428	0.566
Baseline EF<45%	2.965	0.378; 23.28	0.301
Randomized to control	4.267	1.988; 9.158	<0.001

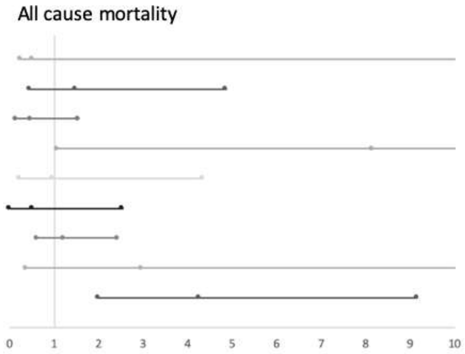

Suppl. Figure S2a

Supplementary Figure S2a. Clinical primary safety endpoint analyses of the ACCRUE IPD patients: all-cause mortality (n=8 studies)

A. Kaplan-Meier analysis of time-dependent all-cause mortality of patients randomized to receive either percutaneous intramyocardial cell-therapy or placebo/medical therapy (controls)

B. Detailed mortality data of the separate studies

C. Hazard ratio and 95% confidence intervals (Cox-regression) of factors support treatment.

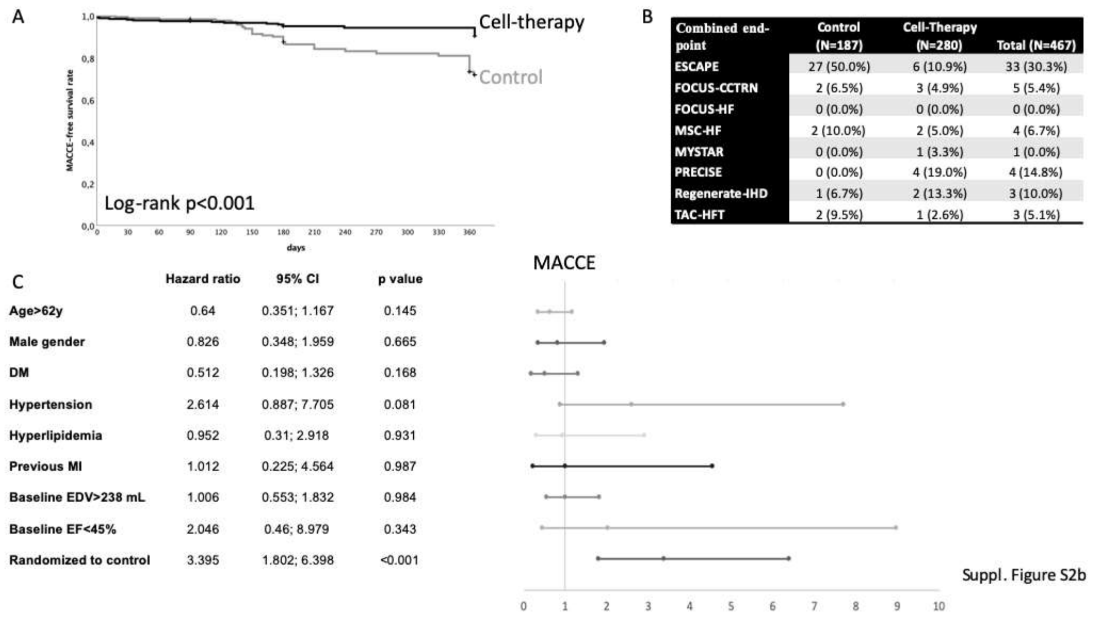

B

Combined end-point	Control (N=187)	Cell-Therapy (N=280)	Total (N=467)
ESCAPE	27 (50.0%)	6 (10.9%)	33 (30.3%)
FOCUS-CCTRN	2 (6.5%)	3 (4.9%)	5 (5.4%)
FOCUS-HF	0 (0.0%)	0 (0.0%)	0 (0.0%)
MSC-HF	2 (10.0%)	2 (5.0%)	4 (6.7%)
MYSTAR	0 (0.0%)	1 (3.3%)	1 (0.0%)
PRECISE	0 (0.0%)	4 (19.0%)	4 (14.8%)
Regenerate-IHD	1 (6.7%)	2 (13.3%)	3 (10.0%)
TAC-HFT	2 (9.5%)	1 (2.6%)	3 (5.1%)

C

	Hazard ratio	95% CI	p value
Age>62y	0.64	0.351; 1.167	0.145
Male gender	0.826	0.348; 1.959	0.665
DM	0.512	0.198; 1.326	0.168
Hypertension	2.614	0.887; 7.705	0.081
Hyperlipidemia	0.952	0.31; 2.918	0.931
Previous MI	1.012	0.225; 4.564	0.987
Baseline EDV>238 mL	1.006	0.553; 1.832	0.984
Baseline EF<45%	2.046	0.46; 8.979	0.343
Randomized to control	3.395	1.802; 6.398	<0.001

Supplementary Figure S2b. Clinical safety secondary endpoint analyses of the ACCRUE patients: MACCE (n=8 studies)

- **A.** Kaplan-Meier curve of time-dependent MACCE (combined endpoint of all-cause death, acute myocardial infarction, stroke, implantation of left ventricular assist device or heart transplantation) of patients randomized to percutaneous intramyocardial cell-therapy or placebo/medical therapy (controls)
- B. Detailed combined endpoint data of the separate studies
- C. Cox regression of the MACCE predictors with the hazard ratio and 95% confidence intervals.

A

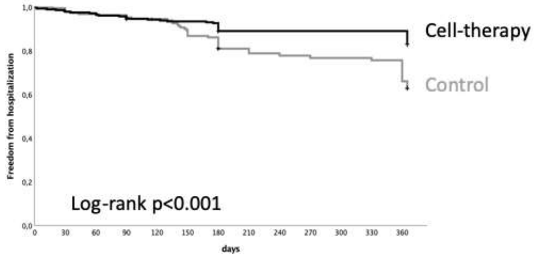

B

Hospitalization	Control (N=187)	Cell-Therapy (N=280)	Total (N=467)
ESCAPE	31 (57.4%)	6 (10.9%)	37 (33.9%)
FOCUS-CCTRN	4 (12.9%)	4 (6.6%)	8 (8.7%)
FOCUS-HF	0 (0.0%)	2 (10.0%)	2 (6.7%)
MSC-HF	8 (40.0%)	9 (22.5%)	17 (28.3%)
MYSTAR	0 (0.0%)	2 (6.7%)	2 (3.3%)
PRECISE	0 (0.0%)	3 (14.3%)	3 (11.1%)
Regenerate-IHD	1 (6.7%)	2 (13.3%)	3 (10.0%)
TAC-HFT	5 (23.8%)	11 (28.9%)	16 (27.1%)

C

	Hazard ratio	95% CI	p value
Age>62y	0.644	0.384; 1.08	0.095
Male gender	1.18	0.506; 2.749	0.701
DM	0.563	0.253; 1.251	0.158
Hypertension	1.187	0.562; 2.504	0.653
Hyperlipidemia	1.895	0.642; 5.593	0.247
Previous MI	1.286	0.378; 4.37	0.687
Baseline EDV>238 mL	1.159	0.695; 1.934	0.572
Baseline EF<45%	2.313	0.548; 9.755	0.254
Randomized to control	2.692	1.592; 4.552	**<0.001**

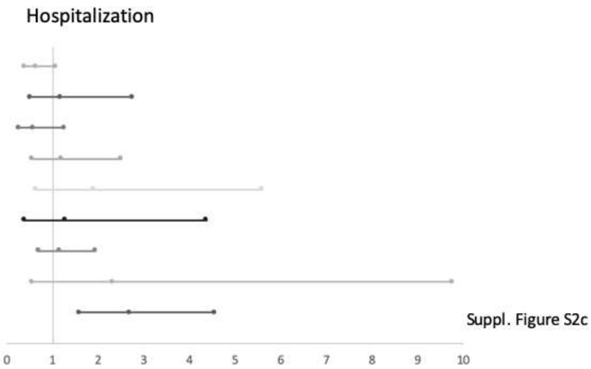

Suppl. Figure S2c

Supplementary Figure S2c. Clinical safety secondary endpoint analyses of the ACCRUE patients: hospitalization (n=8 studies)

A. Freedom from hospitalization during the 1-year follow-up of patients with heart failure and randomized to cell therapy or control.
B. Detailed hospitalization data of the separate studies.
C. Predictors of hospitalization with hazard ratio and confidence intervals

Changes in EF

Study or Subgroup	Cell-therapy Mean	Cell-therapy SD	Cell-therapy Total	Control Mean	Control SD	Control Total	Weight	Mean Difference IV, Random, 95% CI
ESCAPE	4.39	7.17	50	−3.4	8	37	14.3%	7.79 [4.54, 11.04]
FOCUS-CCTRN	1.41	5.19	54	−1.26	5.14	28	16.7%	2.67 [0.32, 5.02]
FOCUS-HF	2.44	6.82	18	4.11	8.92	9	7.3%	−1.67 [−8.29, 4.95]
MSC-HF	4.97	3.83	40	−1.28	3.72	20	17.5%	6.25 [4.23, 8.27]
MYSTAR	3.54	5.58	30	0.1	3.46	30	16.7%	3.44 [1.09, 5.79]
PRECISE	2.28	8.43	16	2.24	6.8	5	6.5%	0.04 [−7.21, 7.29]
REGENERATE-IHD	4.99	8.06	14	4.15	12.27	13	5.8%	0.84 [−7.05, 8.73]
TAC-HFT	0.94	6.03	29	1.29	3.94	17	15.2%	−0.35 [−3.24, 2.54]
**Total (95% CI)**			**251**			**159**	**100.0%**	**3.10 [0.85, 5.34]**

Heterogeneity: $Tau^2 = 6.44$; $Chi^2 = 24.55$, df = 7 (P = 0.0009); $I^2 = 71\%$
Test for overall effect: Z = 2.70 (P = 0.007)

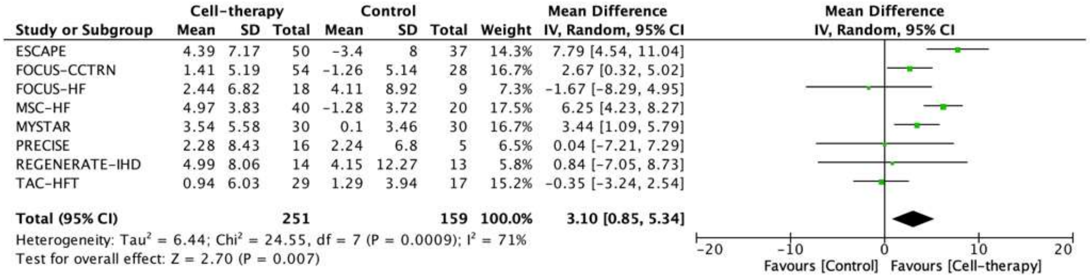

Suppl. Figure S3a

Supplementary Figure S3a. Left ventricular functional primary efficacy analysis of the ACCRUE patients (n=8 studies)

Forest plot of changes in EF from baseline to 1-year follow-up of the groups, favoring cell therapy.

Mean difference between the group was 3.1%, p<0.01.

High heterogeneity between the studies.

Changes in EDV

Study or Subgroup	Favours Cell-therapy Mean	SD	Total	Favours Control Mean	SD	Total	Weight	Mean Difference IV, Random, 95% CI
ESCAPE	-0.3	49.8	50	23.7	39.5	37	15.9%	-24.00 [-42.78, -5.22]
FOCUS-CCTRN	0.9	29.9	54	-8.5	33.8	28	19.0%	9.40 [-5.44, 24.24]
FOCUS-HF	0	0	0	0	0	0		Not estimable
MSC-HF	8.1	19.4	40	2.6	8.7	20	25.3%	5.50 [-1.62, 12.62]
MYSTAR	-2	32.5	30	6.9	40.6	30	16.0%	-8.90 [-27.51, 9.71]
PRECISE	5.6	51.6	16	-28.8	72.4	5	2.5%	34.40 [-33.91, 102.71]
REGENERATE-IHD	3.8	66.7	14	24.1	56.3	13	4.9%	-20.30 [-66.75, 26.15]
TAC-HFT	-1.2	35.6	29	14.4	27.1	17	16.3%	-15.60 [-33.87, 2.67]
**Total (95% CI)**			**233**			**150**	**100.0%**	**-4.74 [-16.09, 6.61]**

Heterogeneity: $Tau^2 = 119.15$; $Chi^2 = 15.49$, df = 6 (P = 0.02); $I^2 = 61\%$
Test for overall effect: Z = 0.82 (P = 0.41)

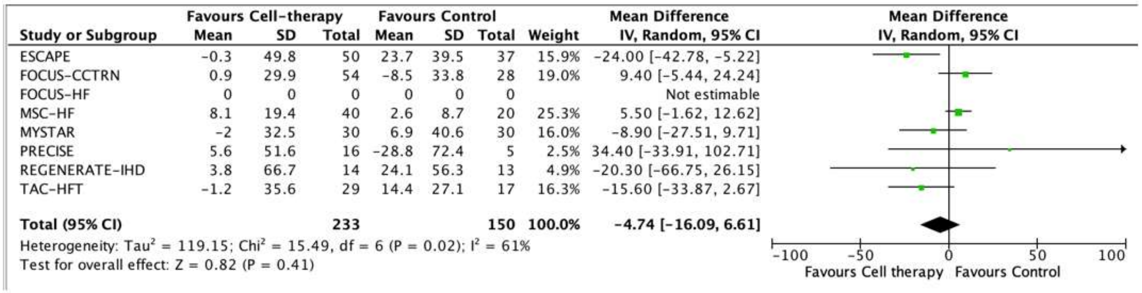

Suppl. Figure S3b

Supplementary Figure S3b. Secondary efficacy endpoint: changes in end-diastolic volume (EDV) of the ACCRUE patients (n=8 studies)

Forest plot of the changes in EDV: no difference between the cell-therapy and controls

Changes in ESV

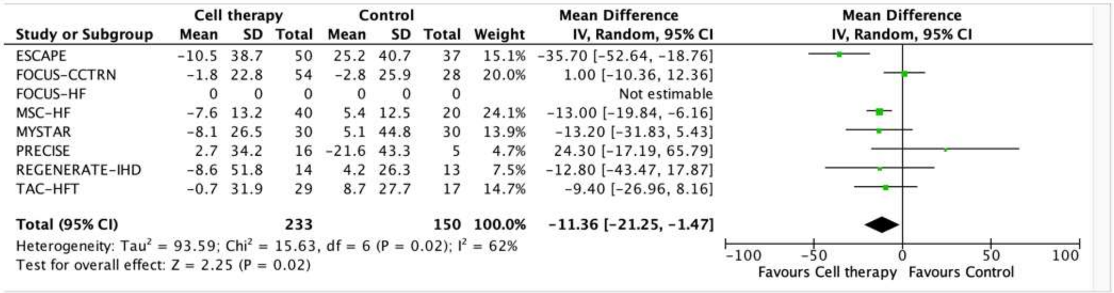

Study or Subgroup	Cell therapy Mean	Cell therapy SD	Cell therapy Total	Control Mean	Control SD	Control Total	Weight	Mean Difference IV, Random, 95% CI
ESCAPE	-10.5	38.7	50	25.2	40.7	37	15.1%	-35.70 [-52.64, -18.76]
FOCUS-CCTRN	-1.8	22.8	54	-2.8	25.9	28	20.0%	1.00 [-10.36, 12.36]
FOCUS-HF	0	0	0	0	0	0		Not estimable
MSC-HF	-7.6	13.2	40	5.4	12.5	20	24.1%	-13.00 [-19.84, -6.16]
MYSTAR	-8.1	26.5	30	5.1	44.8	30	13.9%	-13.20 [-31.83, 5.43]
PRECISE	2.7	34.2	16	-21.6	43.3	5	4.7%	24.30 [-17.19, 65.79]
REGENERATE-IHD	-8.6	51.8	14	4.2	26.3	13	7.5%	-12.80 [-43.47, 17.87]
TAC-HFT	-0.7	31.9	29	8.7	27.7	17	14.7%	-9.40 [-26.96, 8.16]
**Total (95% CI)**			**233**			**150**	**100.0%**	**-11.36 [-21.25, -1.47]**

Heterogeneity: $Tau^2 = 93.59$; $Chi^2 = 15.63$, df = 6 (P = 0.02); $I^2 = 62\%$
Test for overall effect: Z = 2.25 (P = 0.02)

Suppl. Figure S3c

Supplementary Figure S3c. Secondary efficacy endpoint: changes in end-systolic volume (ESV) of the ACCRUE patients (n=8 studies)

Forest plot of the changes in ESV: cell therapy led to significant decrease in ESV as compared to controls

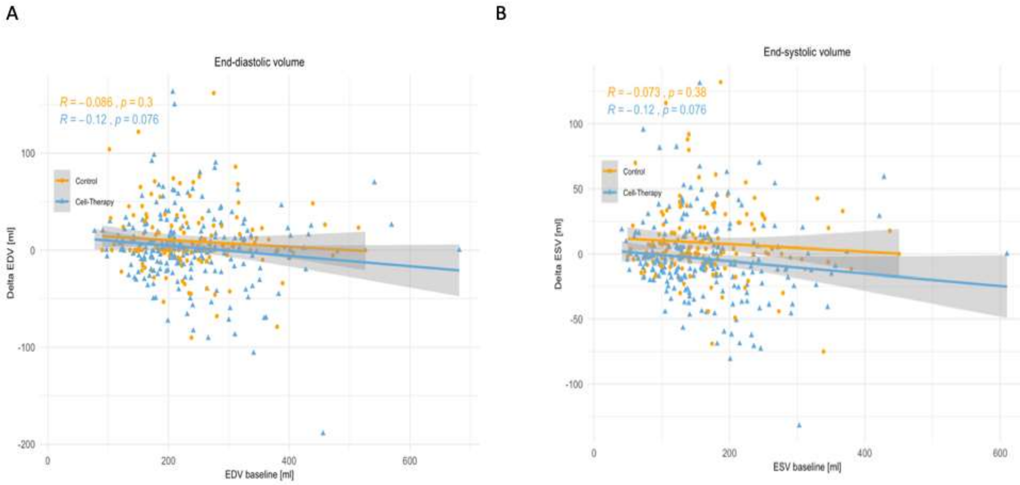

Suppl. Figure S4

Supplementary Figure S4. Association between baseline end-diastolic (EDV) and end-systolic volumes (ESV) and changes in EDV and changes in ESV of the ACCRUE patients

No correlation between baseline EDV and changes in EDV (A) and baseline ESV and changes in ESV (B) in the ACCRUE patients

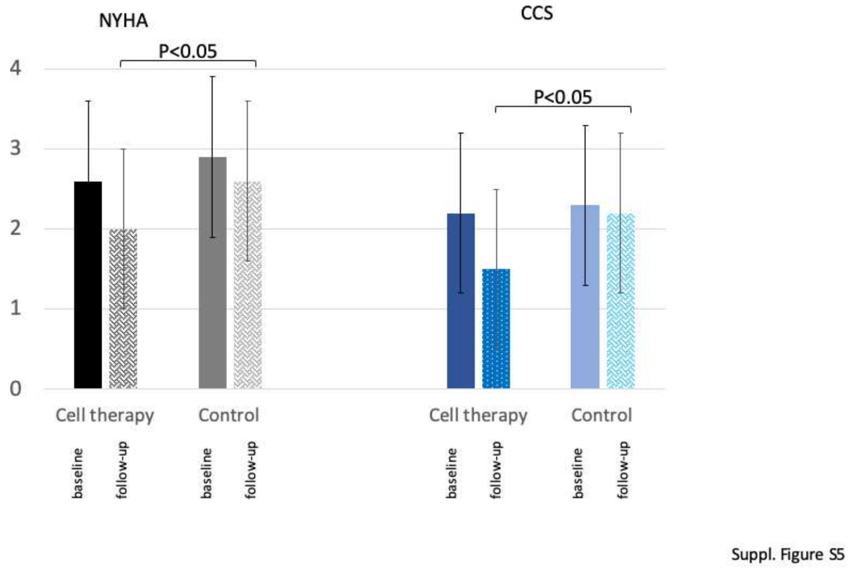

Supplementary Figure S5. Significant improvement in NYHA and CCS classes during the follow-up of the ACCRUE patients

Supplementary References

1. Paitazoglou, C.; Bergmann, M.W.; Vrtovec, B.; Chamuleau, S.A.J.; van Klarenbosch, B.; Wojakowski, W.; Michalewska-Włudarczyk, A.; Gyöngyösi, M.; Ekblond, A.; Haack-Sørensen, M., et al. Rationale and design of the European multicentre study on Stem Cell therapy in IschEmic Non-treatable Cardiac diseasE (SCIENCE). *Eur J Heart Fail* **2019**, *21*, 1032-1041, doi:10.1002/ejhf.1412.
2. Jayaraj, J.S.; Janapala, R.N.; Qaseem, A.; Usman, N.; Fathima, N.; Kashif, T.; Reddy, V.K.; Bakshi, S. Efficacy and Safety of Stem Cell Therapy in Advanced Heart Failure Patients: A Systematic Review with a Meta-analysis of Recent Trials Between 2017 and 2019. *Cureus* **2019**, *11*, e5585, doi:10.7759/cureus.5585.
3. Fan, M.; Huang, Y.; Chen, Z.; Xia, Y.; Chen, A.; Lu, D.; Wu, Y.; Zhang, N.; Qian, J. Efficacy of mesenchymal stem cell therapy in systolic heart failure: a systematic review and meta-analysis. *Stem Cell Research & Therapy* **2019**, *10*, 150, doi:10.1186/s13287-019-1258-1.
4. Fu, H.; Chen, Q. Mesenchymal stem cell therapy for heart failure: a meta-analysis. *Herz* **2020**, *45*, 557-563, doi:10.1007/s00059-018-4762-7.
5. Fisher, S.A.; Doree, C.; Mathur, A.; Taggart, D.P.; Martin-Rendon, E. Stem cell therapy for chronic ischaemic heart disease and congestive heart failure. *Cochrane Database of Systematic Reviews* **2016**, 10.1002/14651858.CD007888.pub3, doi:10.1002/14651858.CD007888.pub3.
6. Fisher, S.A.; Doree, C.; Mathur, A.; Martin-Rendon, E. Meta-Analysis of Cell Therapy Trials for Patients with Heart Failure. *Circulation Research* **2015**, *116*, 1361-1377, doi:10.1161/CIRCRESAHA.116.304386.
7. Tian, T.; Chen, B.; Xiao, Y.; Yang, K.; Zhou, X. Intramyocardial autologous bone marrow cell transplantation for ischemic heart disease: a systematic review and meta-analysis of randomized controlled trials. *Atherosclerosis* **2014**, *233*, 485-492, doi:10.1016/j.atherosclerosis.2014.01.027.
8. Xu, R.; Ding, S.; Zhao, Y.; Pu, J.; He, B. Autologous transplantation of bone marrow/blood-derived cells for chronic ischemic heart disease: a systematic review and meta-analysis. *The Canadian journal of cardiology* **2014**, *30*, 1370-1377, doi:10.1016/j.cjca.2014.01.013.
9. Chun Xiao, S.Z., Yueqiang Liu, Huozhen Hu. Efficacy and Safety of Bone Marrow Cell Transplantation for Chronic Ischemic Heart Disease: A Meta-Analysis. *Med Sci Monit* **2014;**, *20:1768-1777*, doi:10.12659/MSM.892047.
10. Fisher, S.A.; Brunskill, S.J.; Doree, C.; Mathur, A.; Taggart, D.P.; Martin-Rendon, E. Stem cell therapy for chronic ischaemic heart disease and congestive heart failure. *The Cochrane database of systematic reviews* **2014**, 10.1002/14651858.CD007888.pub2, Cd007888, doi:10.1002/14651858.CD007888.pub2.
11. Cheng, K.; Wu, F.; Cao, F. Intramyocardial autologous cell engraftment in patients with ischaemic heart failure: a meta-analysis of randomised controlled trials. *Heart, lung & circulation* **2013**, *22*, 887-894, doi:10.1016/j.hlc.2013.04.112.
12. Kandala, J.; Upadhyay, G.A.; Pokushalov, E.; Wu, S.; Drachman, D.E.; Singh, J.P. Meta-analysis of stem cell therapy in chronic ischemic cardiomyopathy. *The American journal of cardiology* **2013**, *112*, 217-225, doi:10.1016/j.amjcard.2013.03.021.
13. Wen, Y.; Chen, B.; Wang, C.; Ma, X.; Gao, Q. Bone marrow-derived mononuclear cell therapy for patients with ischemic heart disease and ischemic heart failure. *Expert Opinion on Biological Therapy* **2012**, *12*, 1563-1573, doi:10.1517/14712598.2012.721764.
14. Zhao, Q.; Ye, X. Additive value of adult bone-marrow-derived cell transplantation to conventional revascularization in chronic ischemic heart disease: a systemic review and meta-analysis. *Expert Opin Biol Ther* **2011**, *11*, 1569-1579, doi:10.1517/14712598.2011.616491.
15. Donndorf, P.; Kundt, G.; Kaminski, A.; Yerebakan, C.; Liebold, A.; Steinhoff, G.; Glass, A. Intramyocardial bone marrow stem cell transplantation during coronary artery bypass surgery: a meta-analysis. *J Thorac Cardiovasc Surg* **2011**, *142*, 911-920, doi:10.1016/j.jtcvs.2010.12.013.
